# Neuroprotective Capability of Narcissoside in 6-OHDA-Exposed Parkinson’s Disease Models through Enhancing the MiR200a/Nrf-2/GSH Axis and Mediating MAPK/Akt Associated Signaling Pathway

**DOI:** 10.3390/antiox11112089

**Published:** 2022-10-23

**Authors:** Ru-Huei Fu, Chia-Wen Tsai, Shih-Ping Liu, Shao-Chih Chiu, Yen-Chuan Chen, Yu-Ting Chiang, Yun-Hua Kuo, Woei-Cherng Shyu, Shinn-Zong Lin

**Affiliations:** 1 Graduate Institute of Biomedical Sciences, China Medical University, Taichung 40402, Taiwan; 2 Translational Medicine Research Center, China Medical University Hospital, Taichung 40447, Taiwan; 3 Department of Nutrition, China Medical University, Taichung 40402, Taiwan; 4 Buddhist Tzu Chi Bioinnovation Center, Tzu Chi Foundation, Hualien 97002, Taiwan; 5 Department of Neurosurgery, Buddhist Tzu Chi General Hospital, Hualien 97002, Taiwan

**Keywords:** Parkinson disease, narcissoside, 6-hydroxydopamine, SH-SY5Y, *C. elegans*, apoptosis, glutathione, Nrf2, MAPK, miR200a

## Abstract

We assessed the antioxidant potential of narcissoside from *Sambucus nigra* flowers (elderflowers) in Parkinson’s disease models in vitro and in vivo. The results showed that narcissoside lessened the 6-hydroxydopamine (6-OHDA)-induced increase in reactive oxygen species (ROS) and apoptosis in SH-SY5Y cells. In the 6-OHDA-exposed *Caenorhabditis elegans* model, narcissoside reduced degeneration of dopaminergic neurons and ROS generation, and also improved dopamine-related food-sensitive behavior and shortened lifespan. Moreover, NCS increased total glutathione (GSH) by increasing the expression of the catalytic subunit and modifier subunit of γ-glutamylcysteine ligase in cells and nematodes. Treatment with a GSH inhibitor partially abolished the anti-apoptotic ability of narcissoside. Furthermore, narcissoside diminished the 6-OHDA-induced phosphorylation of JNK and p38, while rising activities of ERK and Akt in resisting apoptosis. The antioxidant response element (ARE)-luciferase reporter activity analysis and electromobility gel shift assay showed that narcissoside promotes the transcriptional activity mediated by Nrf2. Finally, we found that narcissoside augmented the expression of miR200a, a translational inhibitor of the Nrf2 repressor protein Keap1. Downregulation of Nrf2 and miR200a by RNAi and anti-miR200a, respectively, reversed the neuroprotective ability of narcissoside. In summary, narcissoside can enhance the miR200a/Nrf2/GSH antioxidant pathway, alleviate 6-OHDA-induced apoptosis, and has the neuroprotective potential.

## 1. Introduction

Parkinson’s disease (PD) is a disorder characterized by the selective death of dopaminergic (DA) neurons in the substantia nigra pars compacta (SN) of the midbrain, commonly seen in older adults. It is closely related to the abnormal increase in reactive oxygen species (ROS) caused by environmental or genetic factors [1,2]. Excessive intracellular ROS and insufficient antioxidants block the function of proteins, nucleic acids and lipids due to abnormal oxidative modification, and finally cause DA neuron damage [3]. In the SN of deceased patients with PD, lipid peroxidation and iron accumulation were observed with reductions in antioxidants such as glutathione (GSH) [4]. Additionally, dopamine undergoes enzymatic oxidation and auto-oxidation, making DA neurons more sensitive to increased ROS levels [5]. Therefore, activation of the antioxidant system in DA neurons in order to reduce the accumulation of ROS and inhibit apoptosis is a current therapeutic strategy for PD.

DA neuron damage caused by 6-hydroxydopamine (6-OHAD) neurotoxin is currently widely used in cell and animal pharmacological models of PD [6]. Studies have confirmed that 6-OHAD can lead to ROS increase, apoptosis, and impairment of cellular functions such as autophagy and the ubiquitin-proteasome system [7]. The main reason is that 6-OHDA specifically enters DA neurons via the dopamine transporter, causing damage to the mitochondrial electron transport chain and inhibiting cellular antioxidant activity, and, finally, raises the production of ROS to develop an oxidative stress [8].

Glutathione (GSH) is the most important antioxidant and redox modulator in cells for ROS elimination and detoxification [9]. Studies have shown that GSH deletion causes oxidative stress-related cellular dysfunction [10]. γ-Glutamylcysteine ligase (γ-GCL), composed of the GCL modifier subunit (GCLM) and GCL catalytic subunit (GCLC), is a key step enzyme in GSH synthesis [11]. The nuclear factor erythroid-2 related factor (Nrf2) is known to bind to the antioxidant responsive element (ARE) in the promoter region of the γ-GCL gene and activate its transcription [12]. Nrf2-mediated ARE activation promotes neuroprotection in cellular and animal models [13].

Mitogen-activated protein kinases (MAPK) of p38 and c-Jun N-terminal kinase (JNK) have been implicated as important signaling molecules for cellular apoptosis induced by excess ROS [14]. Studies have shown that the activity of the JNK and p38 pathways is increased in the DA neurons of patients with PD [15,16]. Exposure of 6-OHDA enhanced the phosphorylation of JNK and p38 in SH-SY5Y cells, resulting in cytochrome c release and caspase 3 activation. Inhibiting these signaling molecules may alleviate apoptosis [17]. Extracellular signal-regulated kinase (ERK) 1/2 and Akt are involved in cellular survival signaling. Disruption of Akt/Erk cascade is significantly associated with the pathogenesis of PD [18].

MicroRNAs (MiR) are single-stranded, non-coding, small RNA molecules [19]. The primary transcript of miRNA is pri-miRNA. After being processed by the Drosha enzyme, it is converted into pre-miRNA with a stem-loop structure and transported outside the nucleus. Finally, it is cleaved by the cytoplasmic Dicer enzyme and combined into the RNA-induced silencing complex (RISC) to develop a functional miRNA. MiRNAs are mainly involved in the regulation of post-transcriptional expression of genes [20]. They inhibit target mRNA translation and expression through complementary bonding [21].

**Figure 1:**
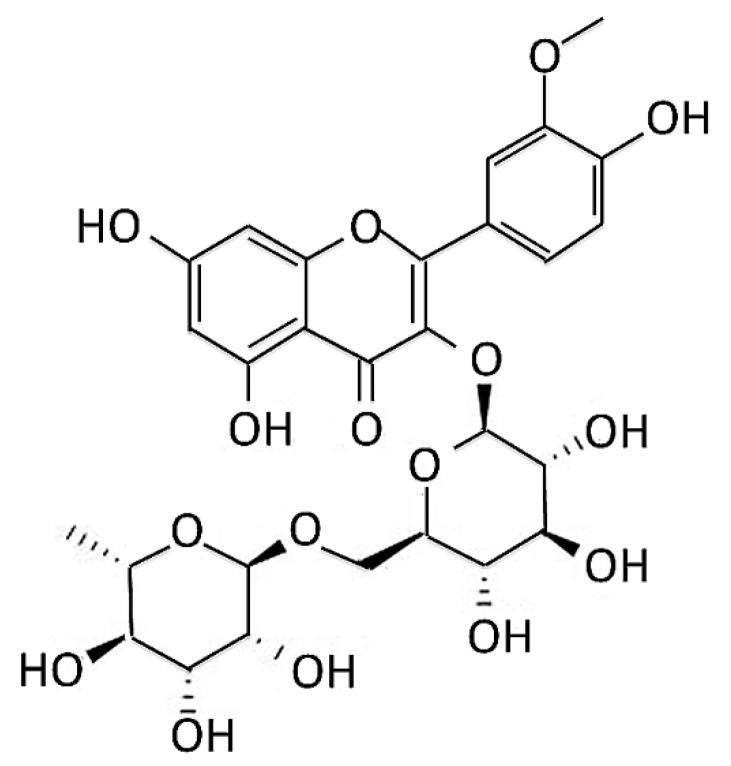
Chemical structure of narcissoside (NCS) from *Sambucus nigra* flowers (elderflowers).

Although few patients’ PD is associated with genetic variants, the exact cause of most PD remains unclear. The development of related drugs is limited to dopamine supplementation and maintenance, alleviation of symptoms or anti-inflammation [22]. The use of special phytochemicals to develop novel anti-oxidative, mitochondrial protection, and anti-apoptotic drugs on DA neurons may achieve the purpose of treating or curing PD. Narcissoside (NCS) (Figure 1) is isolated from *Sambucus nigra* flowers (elderflowers), and has been reported to have several pharmacological activities, such as antioxidant [23,24], anti-inflammatory activities [25], anti-SARS-CoV-2 [26], antihypertensive properties [27], and antitumor potential [28,29]. It also activates TRPV1 channels in dorsal root ganglion sensory neurons linked with pain-accompanied itch [30].

In this study, we used SH-SY5Y cells as an in vitro model and *Caenorhabditis elegans* (*C. elegans*) as an in vivo model to evaluate the neuroprotective properties of NCS in 6-OHDA causing cytotoxicity and to indicate NCS acting mechanisms. *C. elegans* is a facile animal platform for PD-related mechanistic studies and preliminary drug screening. The results demonstrated that NCS can protect SH-SY5Y cells and nematodes against 6-OHDA-induced ROS and apoptosis, in part by enhancing the miR200a/Nrf2/GSH axis and ERK/Akt pathway, while inhibiting the phosphorylation of signaling molecules JNK and p38. Therefore, NCS can be applied to the development of candidate agents for PD therapy in the future.

## 2. Materials and Methods

### 2.1. Narcissoside, Chemicals, Maintenance and Pretreatment of SH-SY5Y Cell Line

Synthesized narcissoside (NCS, mol. wt. 624.54, 98% purity) was obtained from Rainbow Biotechnology Co., Ltd. (Shilin, Taipei, Taiwan) and was prepared to 1-M stock solution by dissolving in DMSO. Other chemicals were acquired from Sigma-Aldrich (St. Louis, MO, USA), unless otherwise stated. SH-SY5Y cell line (ATCC, CRL2266, human neuroblastoma) was a gift from Chia-Wen Tsai (China Medical University, Taichung, Taiwan). Culture medium, supplement and fetal bovine serum (FBS) were obtained from Gibco, ThermoFisher Scientific (Waltham, MA, USA). The cells were cultured in DMEM containing 1.5 g/L sodium bicarbonate, 2 mM L-glutamine, 1.0 mM sodium pyruvate, 0.1-mM nonessential amino acids, 1 × 10^5^ unit/L penicillin, 100 mg/L streptomycin, 10% FBS at 37 °C under humidified atmosphere of 95% air and 5% CO_2_.

With the exception of specific experiments, in general NSC pretreatment SH-SY5Y cells (2.5 × 10^6^) were seeded in 60-mm dishes containing 2-μM NCS for 24 h. Next, it was exposed to 100-μM 6-OHDA for 18 h.

### 2.2. Cell Viability Assay

Cells (4 × 10^3^) were seeded in 96-well cell culture plates and processed as described in Section 2.1. Finally, CellTiter-Blue^®^ Reagent (CellTiter Blue Cell Viability Assay kit, Promega, Madison, WI, USA) was added directly to the cell culture medium and reacted at 37 °C for 2 h, and then the fluorescence signal intensity was measured using SpectraMax M2 Microplate Reader (Molecular Devices, Silicon Valley, CA, USA) (λex = 560; λem = 590 nm).

### 2.3. Mitochondrial Membrane Potential Assay of Cells

Treated cells were washed with PBS, and fresh medium containing 1 μM of 3,3’-dihexyloxacarbocyanine iodide (DiOC6) dye was added. Changes in MMP (green fluorescence) were detected after 30 min using an Axio Observer inverted fluorescence microscope (Carl Zeiss MicroImaging GmbH, Göttingen, Germany) and the fluorescence intensity of the images was quantified using ImageJ software (National Institutes of Health).

### 2.4. Live Cell Nuclear Staining with Hoechst 33258

Treated cells were washed with PBS, and then fresh medium was added. Next, cells were stained with Hoechst 33258 (5 μg/mL) for 1 h at room temperature in the dark. Chromosomal morphology (blue fluorescence) was visualized using an Axio Observer inverted fluorescence microscope (Carl Zeiss MicroImaging GmbH, Göttingen, Germany). The fluorescence intensity of the images was quantified using ImageJ software (National Institutes of Health).

### 2.5. Flow Cytometric Analysis of Apoptosis by FITC-Labeled Annexin-V/Propidium Iodide

We performed apoptosis detection via the FITC Annexin-V Apoptosis Detection Kit I (BD Biosciences Pharmingen, San Diego, CA, USA). Treated cells were washed and resuspended in 100 μL of 1 × binding buffer (10 mM HEPES/NaOH [pH 7.4], 140 mM NaCl, and 2.5 mM CaCl_2_). Next, the FITC-labeled annexin-V and propidium iodide were added to incubate for 15 min in the dark. Finally, 400 μL of 1 × binding buffer was added and analyzed on a BD LSRII flow cytometer (Becton Dickinson, Heidelberg, Germany). The collection gate of a cell is 10,000 events per sample. Among them, Q1 is a dead cell, Q2 is a late apoptotic cell, Q3 is a live cell, and Q4 is an early apoptotic cell. The apoptosis ratio = (Q2 + Q4)/(Q1 + Q2 + Q3 + Q4) × 100%.

### 2.6. Western Blot of Protein Expression

Treated cells were washed with cold PBS, and then lysis buffer (25-mM Tris-HCl, 150 mM NaCl, 10% glycerol, 1% Triton X-100, 2 mM EDTA, 1 mM PMSF, 1 μg/mL aprotinin, 1 μg/mL leupeptin, and phosphatase inhibitor) added to incubate on ice. After 30 min, we conducted centrifuge (14,000× *g*) at 4 °C for 20 min and collected the supernatant and stored it at −80 °C. Total protein concentrations were estimated using a Coomassie Plus Protein Assay Reagent kit (Pierce, Rockford, IL, USA) before conducting Western blotting. We mixed 50 µg of the cell extract with sodium dodecyl sulfate (SDS)-sample buffer, boiled it for 10 min, and loaded it into 7.5%, 10%, or 12.5% SDS-polyacrylamide gel (SDS-PAGE). The proteins were separated by electrophoresis and then transferred to a polyvinylidene fluoride (PVDF) membrane. Next, the primary antibody was added and reacted overnight. Finally, the position and intensity of the indicated proteins were detected using horseradish peroxidase (HRP)-conjugated secondary antibody (PerkinElmer Inc., Boston, MA, USA) by the Amersham enhanced chemiluminescence kit (Amersham Biosciences, Piscataway, NJ, USA) and BioSpectrum imaging system (UVP, Upland, CA, USA). Caspase 3, cleaved caspase 3, poly-ADP ribose polymerase (PARP), cleaved PARP antibodies, GCLC, GCLM, p38, phospho-p38, JNK, phosphor-JNK, Erk1/2, phosphor-Erk1/2, Akt, phosphor-Akt, Nrf1 and Nrf2, were purchased from Cell Signaling Technology (Beverly, MA, USA). Monoclonal antibodies to lamin B1 and β-tubulin were from Santa Cruz Biotechnology, Inc. (Santa Cruz, CA, USA). HRP goat anti-rabbit and HRP goat anti-mouse secondary antibodies were from PerkinElmer, Inc. (Boston, MA, USA).

### 2.7. Quantitative Determination of Intracellular Reactive Oxygen Species

25 μM of 2′,7′-dichlorodihydrofluorescein diacetate (H2DCFDA) was added to treated cells (5 × 10^3^ cells) cultured in black 96-well plates and reacted at 37 °C in the dark for 30 min. Cells were then washed with PBS, and the fluorescence intensity within each well was measured and quantified using a SpectraMax M2 microplate reader (Molecular Devices) (λex = 485; λem = 520 nm). Absorbance was recorded every 15 min for 150 min.

### 2.8. Determines Intracellular Glutathione (GSH) Content

Treated cells were washed with PBS and scraped from the dish. Next, cells were lysed with the MES buffer [0.2 M 2-(N-morpholino)-ethanesulponic acid, 0.05-M phosphate, and 1 mM EDTA, pH 6.0] included in the glutathione detection kit (Cayman Chemical Co., Ann Arbor, MI, USA). The supernatant was then obtained by centrifugation at 10,000× *g* for 15 min at 4 °C and loaded into a 96-well plate. Absorbance was detected and quantified at 405 nm using a SpectraMax M2 microplate reader (Molecular Devices).

### 2.9. Preparation of Nuclear Extract

Treated cells were washed with PBS and centrifuged. The pellet was added to hypotonic buffer (10 mmol/L HEPES, 1 mmol/L MgCl_2_, 10 mmol/L KCl, 0.5 mmol/L DTT, 1 mmol/L EDTA, 20 μg/mL aprotinin, 4 μg/mL leupeptin, 0.5% Nonidet P-40, and 0.2 mmol/L phenylmethylsulfonyl fluoride) and placed on ice for 15 min. Then, the suspension was centrifuged at 6000× *g* for 15 min to acquire crude nuclei. Next, the pellets were resuspended in hypertonic buffer (10 mmol/L HEPES, 1 mmol/L MgCl_2_, 400 mmol/L KCl, 0.5 mmol/L DTT, 1 mmol/L EDTA, 20 μg/mL aprotinin, 4 μg/mL leupeptin, 10% glycerol, and 0.2 mmol/L phenylmethylsulfonyl fluoride) and incubated for 30 min. Finally, the nuclear extracts were acquired by centrifugation at 10,000× *g* for 15 min and were stored at −80 °C.

### 2.10. NRF2/ARE Luciferase Reporter Assays

We generated a repeat of ARE double-stranded oligonucleotide 5′-TGACTCAGCA-3′, spanning the binding site of Nrf2 by gene synthesis (Genewiz, NJ, USA), and cloned it into the promoter region of pGL3 luciferase reporter vectors (Promega), producing p2xARE/Luc plasmid. This reporter system contains the multimerized ARE response element of the promoter and the firefly luciferase gene under its control [31]. We co-transfected cells with 2 μg of p2xARE/Luc and pSV-β-galactosidase control plasmid (Promega) using lipofectamine 2000 reagent (Invitrogen, Carlsbad, CA, USA), according to the manufacturer’s instruction. After 24 h, the cells were replaced with DMEM medium containing serial dilutions of NCS and treated for the indicated times. Next, cells were washed and scraped with a lysis buffer (Promega) and centrifuged at 14,000× *g* for 5 min. Finally, supernatants were collected and luciferase activity was determined using a luciferase assay kit (Promega), according to the manufacturer’s instruction. β-galactosidase activity was detected at 420 nm by O-nitrophenyl-β-D-galactopyranoside (substrate). Each sample was corrected for luciferase activity by β-galactosidase activity.

### 2.11. Electrophoretic Mobility Shift Assay (EMSA)

We used NRF2/ARE Electrophoretic-Mobility Shift Assay (EMSA) Kit (Signosis, Inc., Santa Clara, CA, USA) to assess the ability of NCS to promote the activity of Nrf2 binding to ARE. First, we added 5 µg of nucleoprotein, biotin-labeled double-stranded ARE oligonucleotide, poly(dI-dC) to binding buffer to a final volume of 20 µL, according to the manufacturer’s instruction manual. Next, the mixture was incubated at room temperature for 30 min and separated by electrophoresis on 6% Tris-boric acid-EDTA-polyacrylamide gel. Subsequently, the gel with nucleoprotein-DNA complexes were electro-transferred onto the Hybond-N + nylon membrane (GE Healthcare, Buckinghamshire, UK). Finally, treptavidin-horseradish peroxidase was added to the membranes and incubated for 1 h at room temperature. The nuclear protein-DNA signals were detected using the Amersham-enhanced chemiluminescence kit (Amersham Biosciences) and BioSpectrum imaging system (UVP). 100-fold excess unlabeled double-stranded oligonucleotides (cold) as a competitive group confirmed the specificity of DNA-protein interaction.

### 2.12. Transient Transfection of Small Interfering RNA of Nrf2

Cells were cultured on 35 mm dishes at 80% confluence and then transfected with Nrf2 siRNA (75 nM) or nontargeting control siRNA, using the lipofectamine 2000 reagent (invitrogen), according to the manufacturer’s instructions, for 24 h. The transfected sequences of Nrf2 siRNA were as follows: (1) 5′-CACCUUAUAUCUCGAAGUU-3′, (2) 5′-UAAAGUGGCUGCUCAGAAU-3′.

### 2.13. Measurement of MiR-200a Expression Level by qRT–PCR

We used miRNeasy FFPE Kit 50 (Qiagen, Hilden, Germany) to isolate miRNAs, according to the manufacturer’s instructions. The miScript II Reverse Transcription Kit (Qiagen) reverse transcribed 1 mg of total RNA for detection of miR-200a from cell lines. Quantification of miRNAs was implemented using the miScript SYBR Green PCR kit (Qiagen) and the Hs_miR-200a miScript Primer Assay (mature miRNA sequence: 5′-UAACACUGUCUGGUAACGAUGU-3′, Qiagen), according to the manufacturer’s recommendations on an ABI StepOnePlus system (Applied Biosystems, Inc., Foster City, CA, USA).

### 2.14. Downregulation of miR200a with Anti-miR200a

Anti-miR-200a (miR-200a inhibitor, Qiagen) and miScript inhibitor-negative control (Qiagen) were transiently transfected into SH-SY5Y cells using Lipofectamine 2000 (Invitrogen), according to the manufacturer’s instructions. Anti-miR-200a and miScript inhibitor-negative control were both at an initial concentration of 50 µM, and the AllStars cell death control (Qiagen) was used to confirm the transfection efficiency.

### 2.15. C. elegans Culture and Synchronization

Wild-type Bristol N2 *C. elegans*, transgenic BZ555 strain (Pdat-1::GFP), transgenic N5901 strain (Punc-54::α-Syn::YFP), transgenic DA2123 strain (Plgg-1::GFP::lgg-1), and *Escherichia coli* strains OP50 were provided academically by Caenorhabditis Genetics Center (University of Minnesota, Saint Paul, MN, USA). The general culture and synchronization of the nematodes were performed using a previously described protocol [32].

### 2.16. Food Clearance Assay Determines the Concentration of Narcissoside to Treat Nematodes

Following the food clearance assay [32], we serially diluted NCS into S medium to the indicated concentrations and added overnight cultured OP50 *E. coli* to an optical density (OD) of 6.6. Next, NCS/OP50/S-medium (OD = 0.6, 50 µL) was loaded into a 96-well plate and about twenty L1 nematodes (in 10 µL of S medium) were added. Finally, the OD _595nm_ of the cultures was measured daily for 6 days using a SpectraMax M2 microplate reader (Molecular Devices).

### 2.17. Narcissoside Pretreatment and 6-OHDA Exposure in Nematodes

L1 worms were transferred to a NGM/OP50/NSC medium for 24 h to L3 stage and then exposed to a solution of 50 mM 6-OHDA/10 mM ascorbic acid for 1 h. Finally, nematodes were washed with M9 buffer and then transferred to NSC/OP50/NGM/5-fluoro-2′-deoxyuridine, 2′-deoxy-5-fluorouridine (FUDR, 0.04 mg/mL) medium for culture. The nematodes were used for various experimental analyses after 3 days.

### 2.18. Analysis of Dopaminergic Neuron Degeneration in Nematodes

Treated BZ555 nematodes were washed with M9 buffer, placed on glass slides covered with 2% agar pad, anesthetized with sodium azide (100 mM), and covered with coverslips. In turn, the fluorescence of three pairs of DA neurons in the nematode head was imaged using a Zeiss Axio Imager A1 fluorescence microscope (Carl Zeiss MicroImaging GmbH, Göttingen, Germany) and the fluorescence signal intensities were calculated using ImageJ software (National Institutes of Health) Additionally, we also assessed the degeneration of DA neurons in nematodes by observation. The DA neurons of the nematodes were judged to be degenerated if the dendrites bubbled or disappeared.

### 2.19. Assessment of Dopamine Neuron Functionality in Nematodes with a Food Sensitivity Behavioral Test

In the food perception behavior test [32], we first prepared measurement Petri dishes by smearing OP50 in a 9 cm NGM Petri dish (an inner diameter of 1 cm and an outer diameter of 8 cm), and then the dishes were cultured overnight. Next, the treated N2 nematodes were washed with M9 buffer and placed in the center of the Petri dish. After 5 min, the number of sigmoid movements of each nematode in 20 s on bacterial-free and bacterial lawns were determined. Each nematode was measured 3 times. The slowing rate = (the number of S-shaped movements in the bacteria-free lawn—the number of S-shaped movements in the bacterial lawn)/the number of S-shaped movements in the bacteria-free lawn. Fifty nematodes were assessed for each group.

### 2.20. Lifespan Assessment of Nematodes

In the lifespan assessment of nematodes [32], we transferred the treated L3-stage N2 nematodes to NGM/OP50/FUDR or NGM/OP50/NCS/FUDR plates for culture. Nematodes were transferred to fresh plates every 3 days until the death of 50 nematodes in each group. The number of surviving nematodes was counted daily using a dissecting microscope. Dead worms were defined as unresponsive to repeated contact with the platinum pick-up. Worms that died or disappeared from dehydration by migrating to the wall were excluded from this analysis. Survival curves were obtained using the Kaplan–Meier method and SPSS software (IBM, Armonk, NY, USA).

### 2.21. Determining the Content of Reactive Oxygen Species in Nematodes

According to the Section 2.7, 30 treated nematodes were washed with M9 buffer and then transferred to 96-well plates in 150-μL PBS. Next, 50 μL of H2DCFDA (150 μM in PBS) was mixed and fluorescence was detected using a SpectraMax M2 microplate reader (Molecular Devices). During a 150-min reaction time, the fluorescence value was measured every 15 min.

### 2.22. Determination of Glutathione Content in Nematodes

According to the Section 2.8, 100 treated Nematodes were washed with MES buffer and flash frozen in liquid nitrogen. Then, pellets were ground with pestles in 100 µL of MES buffer and lysed by two freeze-thaw cycles. Next, insoluble material was removed by being centrifuged at 10,000× *g* for 15 min at 4 °C, and supernatant was loaded onto 96-well plate. Absorbance was determined at 405 nm using a SpectraMax M2 Microplate Reader (Molecular Devices). Fluorescence values were corrected for the protein concentration in the lysates (BCA assay).

### 2.23. Total RNA Isolation and Quantitative Analysis of Gene Expression in Nematodes

We extracted the total RNA of nematodes using TRIzol reagent (Invitrogen) and glass beads, according to the previously described method [32]. Quantitation of gene expression used quantitative polymerase chain reaction (qPCR) analysis. We performed qPCR using the SuperScript one-step RT-PCR kit (Invitrogen), SYBR Green I Master kit (Roche Diagnostics, Indianapolis, IN, USA), and ABI StepOnePlus system (Applied Biosystems, Inc.), according to the manufacturer’s instructions. Table 1 lists the primer pairs for this experiment. The analysis was performed using the comparative 2^−ΔΔCt^ method, and the expression of act-1 was used as an endogenous control to calculate the fold difference.

### 2.24. Measurements of the Autophagy Activity of Nematodes

Transgenic DA2123 nematodes, which have a GFP-tagged LGG-1 regulated by the lgg-1 promoter, were used to assess the autophagy activity [31]. The nematodes were washed with M9 buffer, and the LGG-1::GFP-positive puncta (dots) area in the outer epidermal seam cells were detected using a fluorescence microscope. The positive punctate areas of at least 20 seam cells per nematode were calculated. At least 50 nematodes were calculated in each group.

### 2.25. Statistical Analysis of the Investigation

Statistical analysis was employed with commercially obtainable software (SAS Institute Inc., Cary, NC, USA). The experiment was performed at least thrice. Data are expressed as mean ± standard deviation (SD). Statistical significance was determined using one-way ANOVA followed by Tukey’s post hoc test. *p* values < 0.05 were considered to be statistically significant.

## 3. Results

### 3.1. Narcissoside (NCS) Exhibits Protective Potential to Prevent SH-SY5Y Cells from Apoptosis Induced by 6-OHDA Exposure

**Figure 2:**
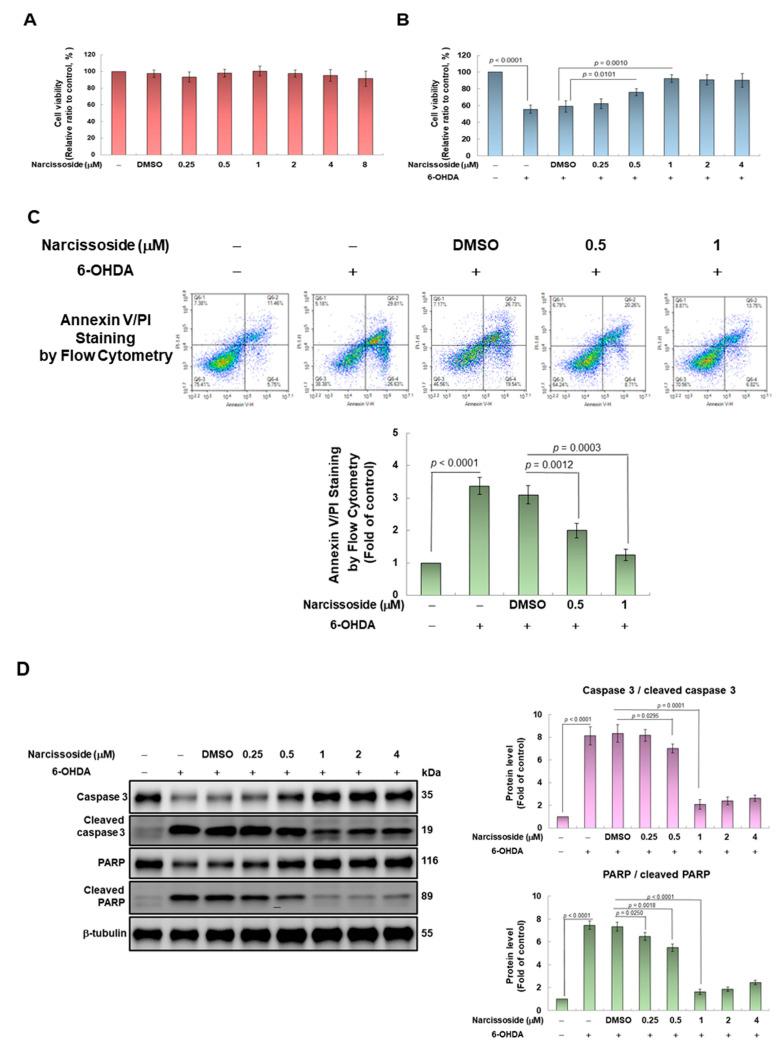
Pretreatment of narcissoside (NCS) attenuated apoptosis of SH-SY5Y cells induced by 6-OHDA exposure. (**A**) Cells were pretreated with the indicated concentrations of NCS for 24 h and then cell viability was measured using CellTiter Blue Cell Viability Assay. The DMSO concentration applied is 0.1%. (**B**) Experimental cells following (**A**) were treated with 100 μM 6-OHDA for an additional 18 h. Cell viability was determined by CellTiter Blue Cell Viability Assay. (**C**) The ratio of cell apoptosis was analyzed using annexin V-FITC binding and propidium iodide (PI) uptake. FITC and PI fluorescence was measured by flow cytometry. (Top). Q1 is a dead cell, Q2 is a late apoptotic cell, Q3 is a live cell, and Q4 is an early apoptotic cell. The apoptosis rate was quantified by (Q2 + Q4)/(Q1 + Q2 + Q3 + Q4) × 100% (bottom) (**D**) The ratio of cleaved caspase 3/pro caspase 3 and cleaved PARP/pro PARP was determined by western blotting and β-tubulin was internal control (left). The intensity of the signal was quantified by the ImageJ software. (right).

First, we used the CellTiter Blue Cell Viability Assay kit to evaluate the toxicity of narcissoside (NCS) to SH-SY5Y cells, and to determine the optimal treatment concentration. The results show that NCS treatment below 8 μM for 24 h did not have a toxic effect on the cells (Figure 2A). Next, we assessed the effect of NCS on cell survival under 6-OHDA exposure. When SH-SY5Y cells were treated to 100 μM 6-OHDA for 18 h, the survival ratio decreased by 44.3% (*p* < 0.0001) in comparison with the control group (Figure 2B). However, under NCS pretreatment for 24 h, the viability of 6-OHDA-exposed cells increased in a dose-dependent manner of NCS. Pretreatment with 1-μM NCS improved the survival rate of 6-OHDA-exposed cells by 1.6-fold compared to the unpretreated group (*p* = 0.0010) (Figure 2B). The protective effect of NCS on cell death induced by 100-μM 6-OHDA was not significantly increased under pretreatments above 2 μM. Therefore, the NCS concentration of subsequent-pretreated cells was up to 1 μM.

Furthermore, we used Annexin V-FITC and PI double staining combined with flow cytometry to evaluate the apoptosis of cells in each group. Results showed that 6-OHDA treatment increased the ratio of apoptotic cells by 3.4 folds (*p* < 0.0001) compared to the control group (Figure 2C). However, NCS dose-dependently reduced the ratio of apoptosis cells in 6-OHDA-exposed cells. Pretreatment with 1 μM NCS significantly declined the ratio of apoptosis cells in 6-OHDA-exposed cells by 61.3% (*p* = 0.0003) compared with the unpretreated group. (Figure 2C).

We also analyzed the effect of NCS pretreatment on the expression of apoptosis-related proteins induced by 6-OHDA via western blotting. We found that 6-OHDA exposure for 12 h augmented the ratio of cleaved caspase 3/pro caspase 3 and cleaved poly (ADP-ribose) polymerase (PARP)/pro PARP in SH-SY5Y cells by 8.1-fold (*p* < 0.0001) and 7.5-fold (*p* < 0.0001), respectively, compared with the control group (Figure 2D). Pretreatment with 1-μM NCS significantly lessened the ratio of cleaved caspase 3/pro caspase 3 and cleaved PARP/pro PARP by 74.7% (*p* = 0.0001) and 78.0% (*p* < 0.0001), respectively, in 6-OHDA exposed cells, compared with the unpretreated group. The above results showed that NCS pretreatment could effectively prevent SH-SY5Y cell apoptosis induced by 6-OHDA exposure.

### 3.2. Pretreatment of Narcissoside (NCS) Enhanced Level of Intracellular Glutathione (GSH) to Diminish Production of Reactive Oxygen Species (ROS) in 6-OHDA-Exposed SH-SY5Y Cells

**Figure 3:**
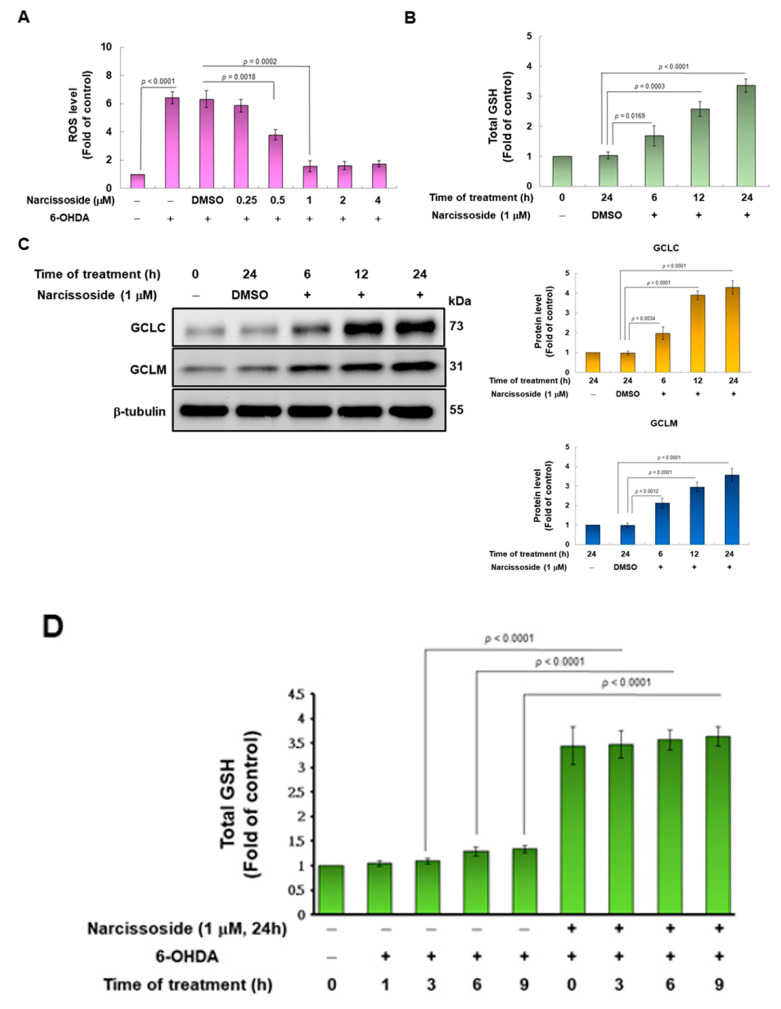

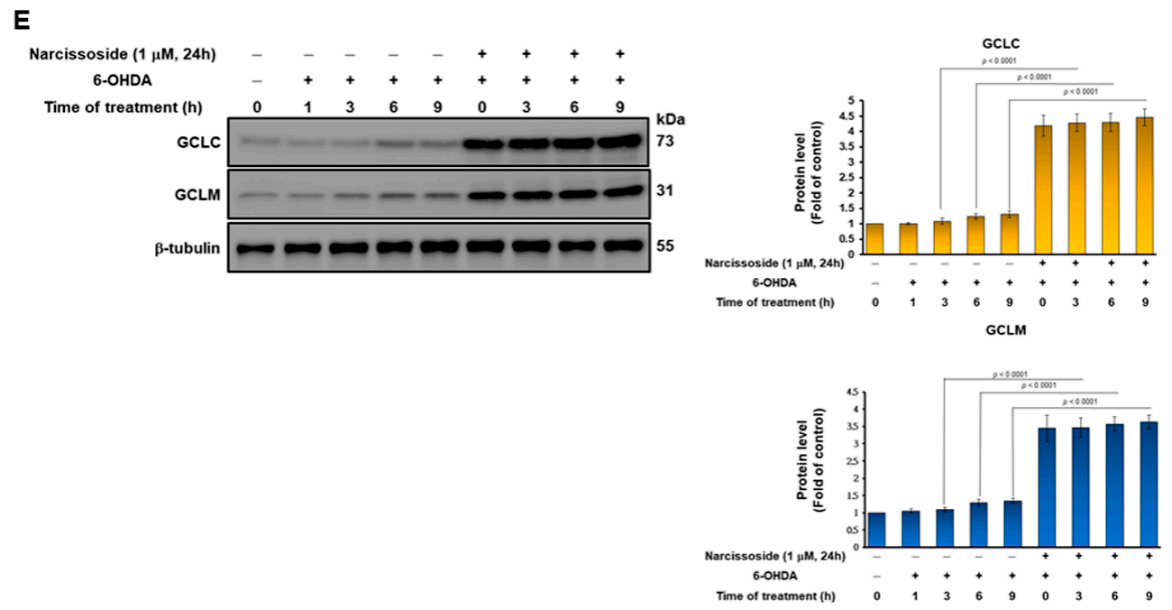
Glutathione (GSH) is involved in the neuroprotective effect of narcissoside (NCS), preventing 6-OHDA-induced reactive oxygen species (ROS) generation in SH-SY5Y cells. (**A**) Cells were pretreated with a series concentration of NCS for 24 h, then treated 100-μM 6-OHDA for 18 h. ROS levels were measured using the H2DCFDA method. (**B**) Cells were treated with 1 μM NCS for 0, 6, 12, or 24 h and then measure GSH level by GSH assay kit. (**C**) The protein expression of the catalytic subunit (GCLC) and modifier subunit (GCLM) of glutamate cysteine ligase (γ-GCL) in (**B**) was determined by western blotting. The expression of β-tubulin is internal control. (left). The intensity of the signal was quantified by the ImageJ software. (right). (**D**) Cells were pretreated with or without 1-μM NCS for 24 h, and then treated 100-μM 6-OHDA for the indicated times. GSH levels were measured using a GSH assay kit. (**E**) The protein expression of GCLC and GCLM in (**D**) was determined by western blotting. The expression of β-tubulin is internal control. (left). The intensity of the signal was quantified by the ImageJ software. (right).

Since 6-OHDA impairs mitochondrial metabolism and the function of the electron transport chain, SH-SY5Y cells produce excess reactive oxygen species (ROS) that damage cells. Therefore, we wanted to use the fluorescent *sensor* probe H2DCFDA to detect the amount of intracellular ROS to evaluate the antioxidant capacity of NCS. The results showed that exposure of cells to 100 μM 6-OHDA augmented ROS production up to 6.4-fold compared to the control group (*p* < 0.0001) (Figure 3A). However, pretreatment with 1 μM NCS significantly reduced intracellular ROS by 74.6% (*p* = 0.0002) in the 6-OHDA-exposed cells (Figure 3A).

The metabolism of intracellular ROS can be converted into less toxic molecules by antioxidants or enzymes, and glutathione (GSH) is a major member. Therefore, we wanted to assess whether NCS would regulate the amount of GSH in SH-SY5Y cells. The results indicated that treatment of 1μM NCS for 24 h raised the amount of GSH by 3.4-fold, compared with the control group (*p* < 0.0001) (Figure 3B). Next, we considered whether the augmented amount of GSH was related to improved expression of the rate-limiting enzyme glutamate cysteine ligase (GCL) at its synthetic step. GCL consists of the catalytic subunit GCLC and the modifier subunit GCLM. The effect of NCS on the expression of GCLC and GCLM was analyzed by western blotting. The results revealed that treatment of 1 μM NCS for 24 h increased the amount of GCLC and GCLM 4.3-fold (*p* < 0.0001) and 3.6-fold (*p* = 0.0001), respectively, compared to the control group (Figure 3C). Whereas, for cells treated to 100-μM 6-OHDA for 9 h, pretreatment with 1-μM NCS for 24 h significantly increased the level of intracellular GSH by 2.8-fold (*p* < 0.0001), compared with the unpretreated group (Figure 3D). The influence of NCS on GCLC and GCLM expression in cells exposed to 6-OHDA for 9 h was analyzed by western blotting. The results showed that pretreatment of 1-μM NCS for 24 h increased the expression of GCLC and GCLM by 3.5-fold (*p* < 0.0001) and 2.8-fold *(p* < 0.0001), respectively, compared with the unpretreated group (Figure 3E).

### 3.3. Treatment with the GSH Synthetic Inhibitor Buthionine Sulphoximine (BSO) Abrogates the Neuroprotective Ability of Narcissoside (NCS) in 6-OHDA-Treated SH-SY5Y Cells

**Figure 4:**
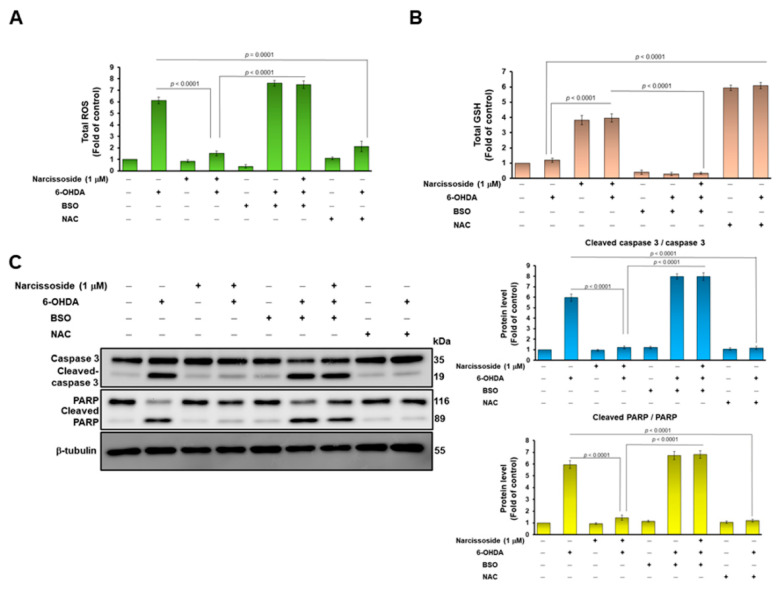
Treatment with the GSH inhibitor butthionine sulfoximine (BSO) reversed the ability of narcissoside (NCS) to inhibit 6-OHDA-induced ROS production and expression of apoptosis-related proteins in SH-SY5Y cells. BSO (100 μM) or NAC (1 mM) was added to SH-SY5Y cells 1 h before NCS pretreatment. Then, cells were incubated with NCS (1 μM) for 24 h, followed by treating them with 100 μM 6-hydroxydopamine (6-OHDA) for 12 h. (**A**) ROS levels were measured using the H2DCFDA method. (**B**) GSH levels were determined by GSH assay kit. (**C**) The ratio of cleaved caspase 3/pro caspase 3 and cleaved PARP/pro PARP was determined by western blotting. The expression of β-tubulin is internal loading control (left). The intensity of the signal was quantified by the ImageJ software. (right).

To confirm the involvement of GSH in the reduction in 6-OHDA-induced ROS by NCS, we used the GSH synthesis inhibitor butthionine sulfoximine (BSO). The results showed that BSO treatment abolished the ability of NCS to suppress ROS production (*p* < 0.0001, Figure 4A) and to promote GSH production (*p* < 0.0001, Figure 4B) in 6-OHDA-exposed SH-SY5Y cells. Furthermore, we used western blotting to analyze the expression of apoptosis-related proteins. Results revealed that BSO treatment reverses the ability of NCS to inhibit caspase 3 and PARP activity in 6-OHDA-exposed SH-SY5Y cells (Figure 4C). Moreover, *N*-*acetylcysteine*, a synthetic precursor of GSH, could promote GSH synthesis (*p* < 0.0001, Figure 4B) to prevent ROS production (*p* = 0.0001, Figure 4A), as well as inhibit caspase 3 (*p* < 0.0001) and PARP activity (*p* < 0.0001) (Figure 4C). Therefore, the BSO effect confirmed that the production of GSH in SH-SY5Y cells is a key event in the inhibition of 6-OHDA-induced apoptosis by NCS.

### 3.4. The Ability of Narcissoside (NCS) to Alleviate 6-OHDA-Induced Apoptosis of SH-SY5Y Cells May Be through Inhibition of p38 and JNK1/2 Mitogen-Activated Protein Kinase (MAPK) Phosphorylation and Enhancing of the Extracellular-Regulated Kinase (ERK) and Akt Pathway

**Figure 5:**
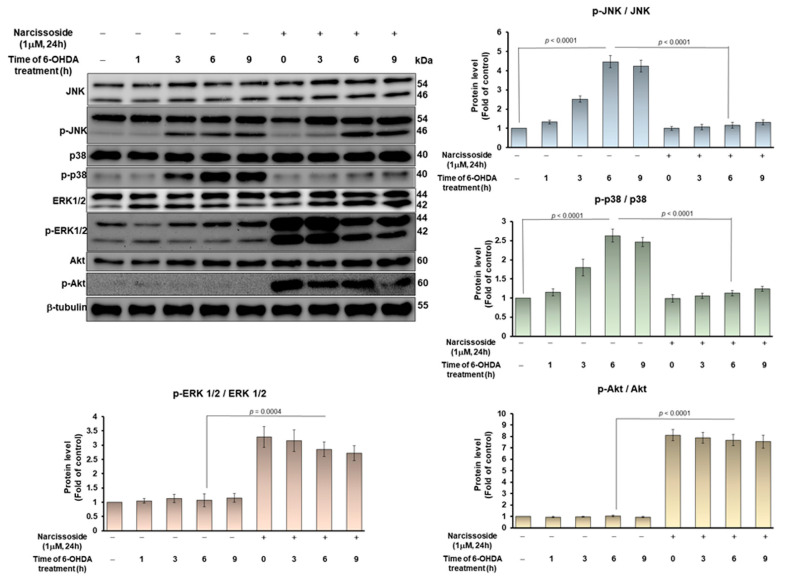
Narcissoside (NCS) attenuated 6-hydroxydopamine (6-OHDA)-induced phosphorylation of JNK and p38, but enhanced ERK1/2 and Akt activity in SH-SY5Y cells. Cells were pretreated with or without 1μM NCS for 24 h and then treated with 100-μM 6-OHDA for 3, 6, and 9 h. Protein levels of JNK, p-JNK, p38, p-p38, ERK1/2, p-ERK1/2, Akt and p-Akt were determined by western blotting. The expression of β-tubulin is as internal loading control. (top left). The intensity of the signal was quantified by the ImageJ software. (right and bottom).

Studies have indicated that 6-OHDA-induced apoptosis of SH-SY5Y cells is related to the activation of JNK and p38 signaling molecular [17], however, the up-regulation of ERK and Akt pathways can prevent this effect [33]. SH-SY5Y cells were pretreated with or without 1-μM NCS for 24 h, and then exposed to 6-OHDA for 3, 6, or 9 h, respectively. As shown in Figure 5, we found that 6-OHDA exposure induced a 4.5-fold (*p* < 0.0001) and a 2.6-fold (*p* < 0.0001) increase in the activity (phosphorylation) of JNK and p38 at 6 h, respectively, compared to the control group. However, the activities of ERK1/2 and AKT did not change significantly. Pretreatment of cells with NCS dose-dependently attenuated 6-OHDA-induced activation of JNK and p38. After exposure of NCS-pretreated cells to 6-OHDA for 6 h, the activation of JNK and p38 was lessened by 73.8% (*p* < 0.0001) and 57.7% (*p* < 0.0001), respectively, compared to the unpretreated group (Figure 5). Conversely, the phosphorylation of ERK1/2 and Akt was increased by 2.6-fold (*p* = 0.0004) and 7.7-fold (*p* < 0.0001), respectively, compared with the unpretreated group (Figure 5).

### 3.5. The Neuroprotective Property of Narcissoside (NCS) in 6-OHDA-Exposed SH-SY5Y Cells Is Associated with the Elevation of the Activity of the Nuclear Factor Erythroid 2–Related Factor 2 (Nrf2) Pathway

**Figure 6:**
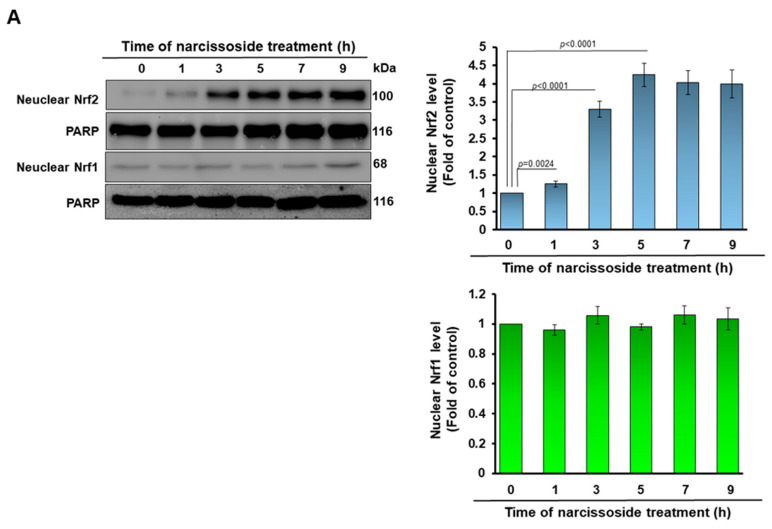

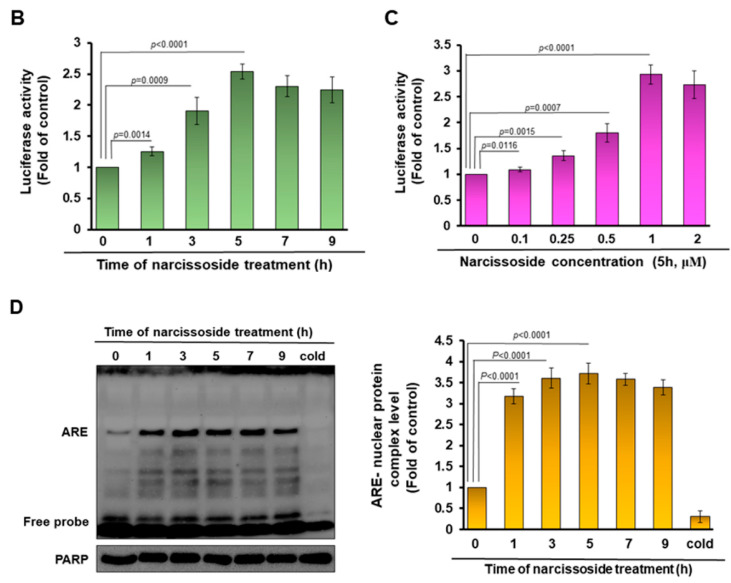
Treatment with narcissoside (NCS) raises the amount of nuclear factor erythroid 2–related factor 2 (Nrf2) in the nucleus of SH-SY5Y cells to enhance the transcriptional activity initiated by binding of the antioxidant response element (ARE). (**A**) Cells were incubated with 1-μM NCS for 0, 1, 3, 5, 7, and 9h. Nuclear Nrf1 and Nrf2 was determined by Western blotting. PARP was used as the internal loading control. (**B**,**C**) Cells were transfected with the ARE reporter plasmid for 24 h and then treated with 1 μM NCS for 0, 1, 3, 5, 7, or 9 h (**B**) or treated 0, 0.1, 0.25, 0.5, or 1 μM NCS for 5 h (**C**). Luciferase activities were determined by Luciferase assay kit. (**D**) Electromobility gel shift assay (EMSA) was used to quantify the ARE binding activity of nuclear extracts (Nrf2) from (**A**). Unlabeled double-stranded ARE oligonucleotide (200 ng, cold competitors) was used to confirm the specific binding.

The binding of the nuclear factor erythroid 2–related factor 1 (Nrf1) and Nrf2 transcription factor with the antioxidant response element (ARE) has been revealed to promote gene transcription of antioxidant or detoxification enzymes such as γ-GCL [34]. Western blotting indicated that nuclear translocation of Nrf2 in SH-SY5Y cells after 5 h of NCS (1 μM) treatment reached the highest; it was 4.2-fold (*p* < 0.0001), and then declined slightly in comparison with the untreated group (Figure 6A). However, the level of Nrf1 in the nucleus did not increase through NCS treatment, indicating its inactivation (Figure 6A). This result is also consistent with the increase in expression of NCS-induced GCLC and GCLM (Figure 3C). Furthermore, NCS dose-dependently increased the Nrf2/ARE-related luciferase activity of SH-SY5Y cells by transient transfection of 2 × ARE reporter (Figure 6B,C). Pretreatment of 1-μM NCS for 5 h increased luciferase activity up to 2.9-fold (*p* < 0.0001) compared to the untreated group (Figure 6C). We further demonstrated by the electrophoretic mobility shift assay (EMSA) that NCS raised the expression of γ-GCL mediated by ARE by increasing the amount of Nrf2 in the nucleus. The results showed that the binding of ARE-DNA repeats to nuclear proteins reached a maximum at 5 h after 1-μM NCS treatment, a 3.7-fold (*p* < 0.0001) increase compared to the untreated group (Figure 6C).

### 3.6. Knockdown of Nrf2 Abolished the Neuroprotective Ability of Narcissoside (NCS) against Oxidative Stress and Apoptosis of SH-SY5Y Induced by 6-OHDA Exposure

**Figure 7:**
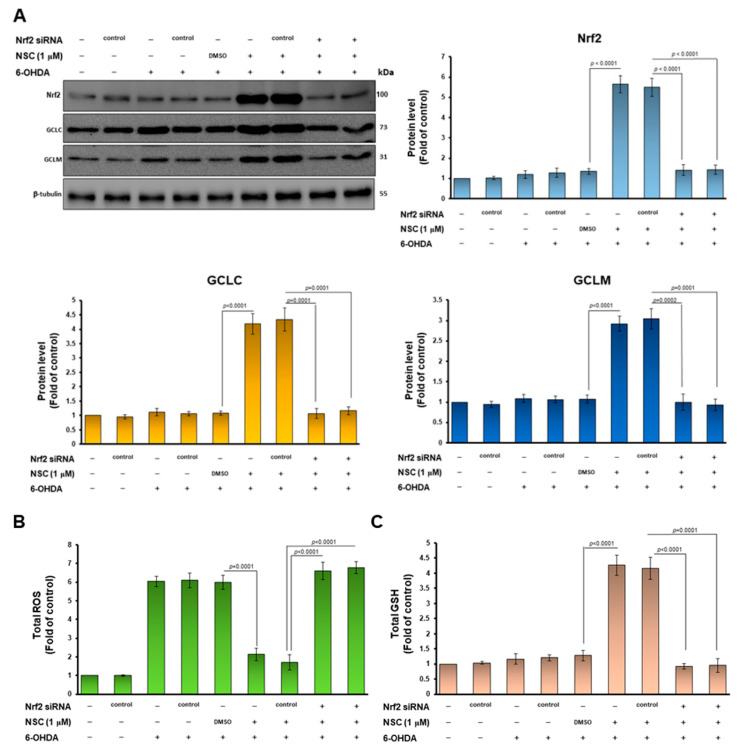

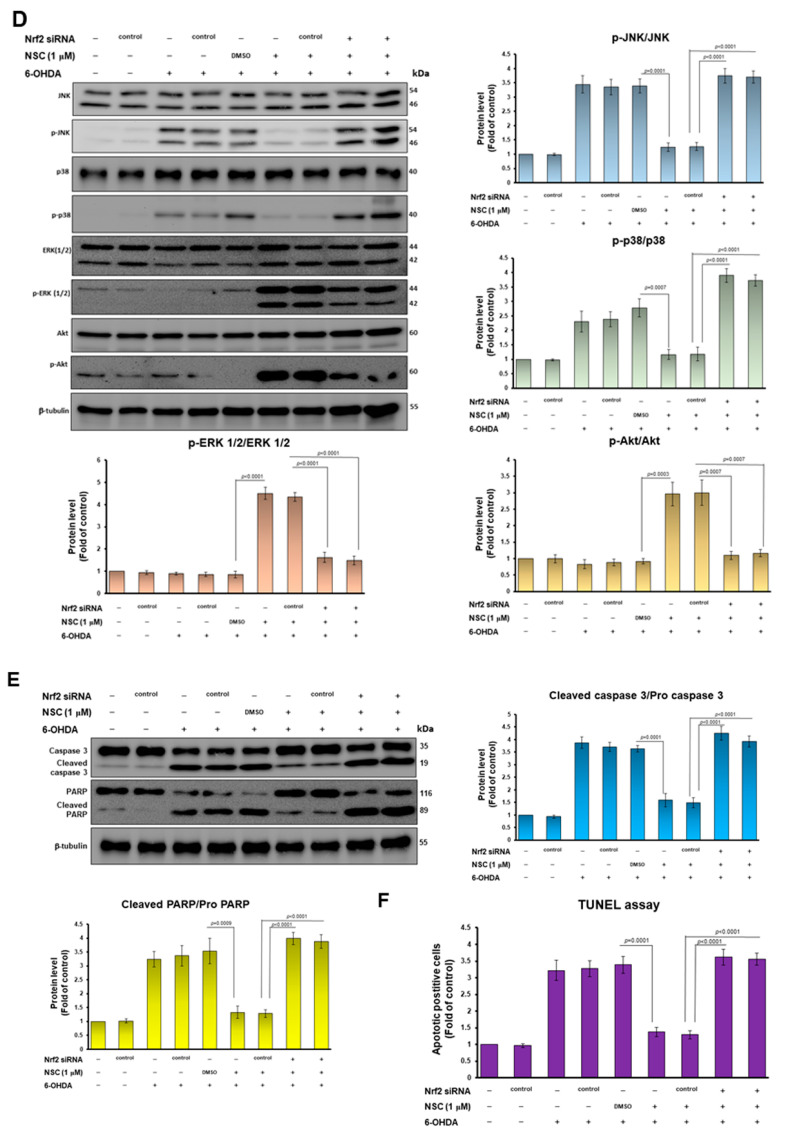
Downregulation of Nrf2 expression in the 6-OHDA-exposed SH-SY5Y cells abrogates the neuroprotective ability of narcissoside (NCS). SH-SY5Y cells were transfected with Nrf2 siRNA or control siRNA for 24 h. It was then pretreated with NCS for 24 h, followed by treatment with 6-OHDA for 6 h. (**A**) The levels of Nrf2, GCLC, and GCLM were analyzed by western blotting. The expression of β-tubulin is internal loading control (top left). The intensity of the signal was quantified by the ImageJ software (right and bottom). (**B**) ROS levels were measured using the H2DCFDA method. (**C**) GSH levels were determined by GSH assay kit. (**D**) Protein levels of JNK, p-JNK, p38, p-p38, ERK1/2, p-ERK1/2, Akt and p-Akt were determined by western blot. The expression of β-tubulin is as internal loading control. (top left). The intensity of the signal was quantified by the ImageJ software. (right and bottom). (**E**) The ratio of cleaved caspase 3/pro caspase 3 and cleaved PARP/pro PARP was determined by western blotting. The expression of β-tubulin is internal loading control (top left). The intensity of the signal was quantified by the ImageJ software. (right and bottom). (**F**) The ratio of apoptotic cells was determined by TUNEL assay.

To confirm that the Nrf2 pathway is involved in the increase in GSH levels, and in the inhibition of the apoptosis signaling pathway in 6-OHDA-exposed SH-SY5Y cells, we performed Nrf2 knockdown experiments by siRNA transfection. The western blotting displayed that Nrf2 siRNA treatment significantly lessened Nrf2 levels by 74.5% (*p* < 0.0001) in the NSC-pretreated 6-OHDA-exposed SH-SY5Y cells compared with the control siRNA group (lane 9, Figure 7A). Furthermore, Nrf2 downregulation abolished the ability of NCS to induce GCLC and GCLM expression in the 6-OHDA-exposed SH-SY5Y cells (Figure 7A). Therefore, Nrf2 RNAi reversed ROS lessening (Figure 7B) and GSH increasing (Figure 7C) in the NSC-pretreated 6-OHDA-exposed SH-SY5Y cells. In the apoptosis signaling, Nrf2 downregulation abolished the ability of NCS to inhibit the phosphorylation of JNK and p38 in the 6-OHDA-exposed SH-SY5Y cells by western blotting (Figure 7D). In the survival pathway, Nrf2 RNAi reversed the NCS-induced ERK1/2 and Akt activity in the 6-OHDA-exposed SH-SY5Y cells (Figure 7D). Moreover, blocking Nrf2 expression invalidates NCS-suppressing caspase 3, PARP activity (Figure 7E) and apoptosis (Figure 7F) in the 6-OHDA-exposed SH-SY5Y cells. This result confirms that the modulation of Nrf2 activity is the primary way in which NCS exhibits its neuroprotective utility.

### 3.7. Narcissoside (NCS) Inhibits Keap1 Level by Augmenting Expression of Endogenous MicroRNA-200a and Thus Enhances Nrf2 Activity

**Figure 8:**
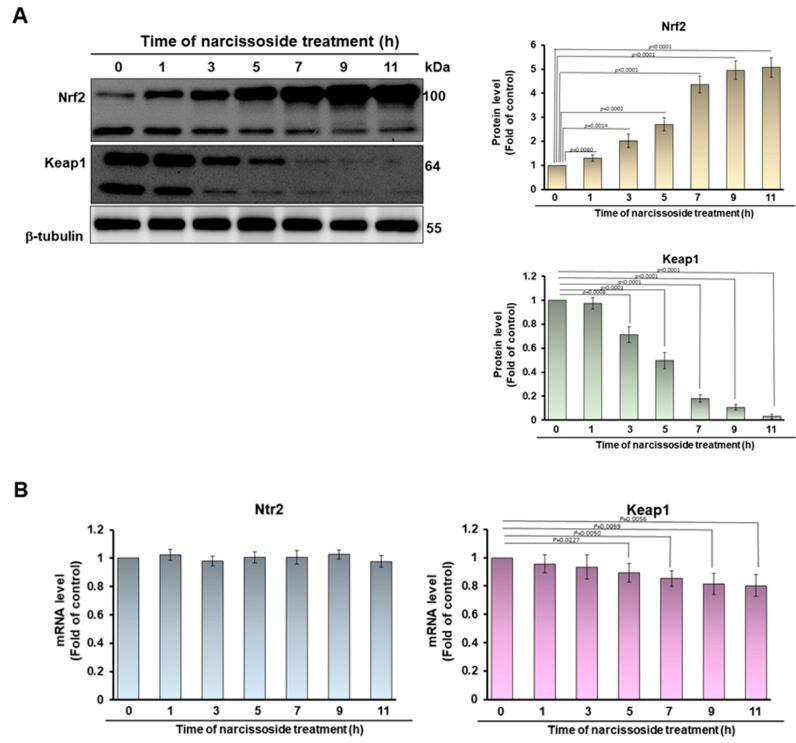

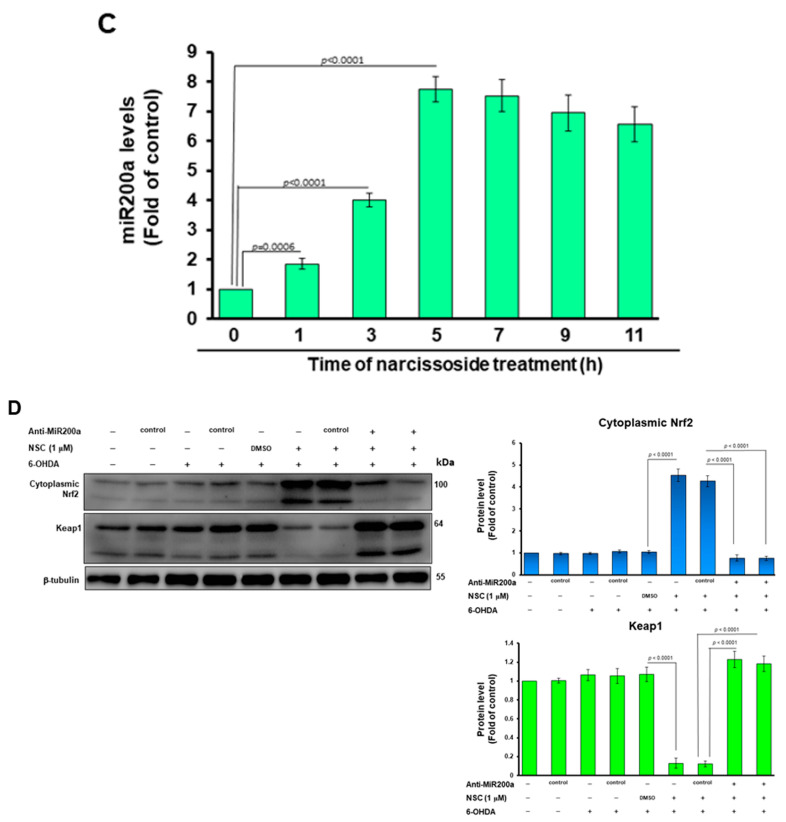

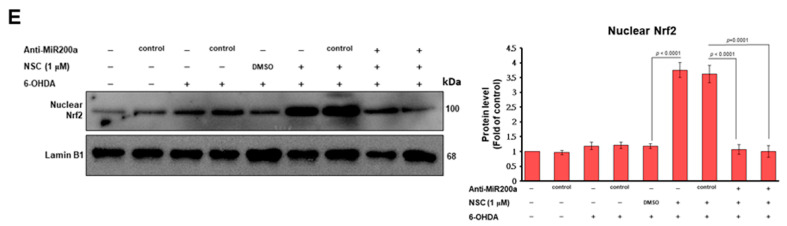
NCS enhances the expression of miR-200a targeting Keap1 to activate the Nrf2 antioxidant pathway in 6-OHDA-exposed SH-SY5Y cells. (**A**) The expression of Nrf2 and its inhibitory protein Keap1 in the cytoplasm of NCS-treated SH-SY5Y cells was observed by Western blotting. B-tubulin is internal loading control (left). The intensity of the signal was quantified by the ImageJ software. (right). (**B**) q-PCR analysis of mRNA expression levels of Ntr2 and Keap1 in SH-SY5Y cells with NSC treatment. (**C**) qRT–PCR analysis of changes in the level of miR200a in SH-SY5Y cells treated with NSC. (**D**) Using anti-miR200a (miR200a inhibitor) to confirm the role of miR200a in the Nrf2 pathway activated by NSCs. Western blotting analyzed the expression of cytoplasmic keap1 and Nrf2 after anti-miR200a transfection in NSC-pretreated 6-OHDA exposed SH-SY5Y cells. The expression of β-tubulin is internal loading control (left). The intensity of the signal was quantified by the ImageJ software. (right). (**E**) Western blotting analyzed the expression of nuclear Nrf2 after anti-miR200a transfection in NSC-pretreated 6-OHDA exposed SH-SY5Y cells. The expression of lamin B1 is internal loading control (left). The intensity of the signal was quantified by the ImageJ software. (right).

To confirm the possible mechanism by which NCS induces Nrf2 activity, we first used Western blotting to observe the cytoplasmic expression of Nrf2 and its repressor Keap1 alter NCS treatment. The results showed that the level of Nrf2 was significantly increased after NCS treatment, while the level of Keap1 was significantly decreased. After 11 h of NSC treatment, Nrf2 was increased by 5.1-fold (*p* < 0.0001) and keap1 was decreased by 97% (*p* < 0.0001), compared with the 0-h group (Figure 8A). Furthermore, we found that the ability of Nrf2 to interact with Keap1 was not significantly altered by NCS treatment using a yeast two-hybrid-based growth assay [35] (data not shown). Next, we used q-PCR to quantify the mRNA expression of Nrf2 and keap1 after NSC treatment. Subsequently, we used q-PCR to quantify the mRNA expression of Nrf2 and Keap1 after NSC treatment. The results showed that the level of Nrf2 mRNA did not change significantly after NCS treatment for 11 h (Figure 8B, left), however, the mRNA expression of Keap1 was significantly lessened, by 19.3%, compared with 0-h group (*p* = 0.0056, Figure 8B, right). Multiple endogenous microRNAs (miR) regulate the expression of Keap1 by blocking translation or promoting degradation. We hypothesized that NSC regulates the expression of specific Keap1-targeting miRs. Q-RT-PCR results showed that the expression level of miR200a was significantly increased after NSC treatment (Figure 8C). Five hours after NCS treatment, the expression of miR200a increased 7.7-fold compared with the 0-h group, and this is consistent with the reduction in Keap1 protein expression (Figure 8A). To confirm the role of miR200a in the Nrf2 pathway activated by NSCs, we used anti-miR200a (miR200a inhibitor), a single-stranded oligonucleotide that specifically binds and inhibits endogenous miR200a. Western blotting showed that anti-miR200a transfection abrogated the NSC-caused decrease in Keap1 protein expression, and the increase in cytoplasmic Nrf2 protein expression in 6-OHDA exposed SH-SY5Y, compared with the anti-miR control group (Figure 8D). Furthermore, the NCS-induced increase in the level of Nrf2 in the nucleus was also reversed by anti-miR-200a treatment. (Figure 8E). These data showed that the role of MiR-200a is a more upstream event in NSC-mediated Nrf2 activity.

### 3.8. Narcissoside (NCS) Pretreatment Was Effective in Reducing Dopamine Neuron Degeneration Induced by 6-OHDA Exposure in an Animal Model of Caenorhabditis elegans

**Figure 9:**
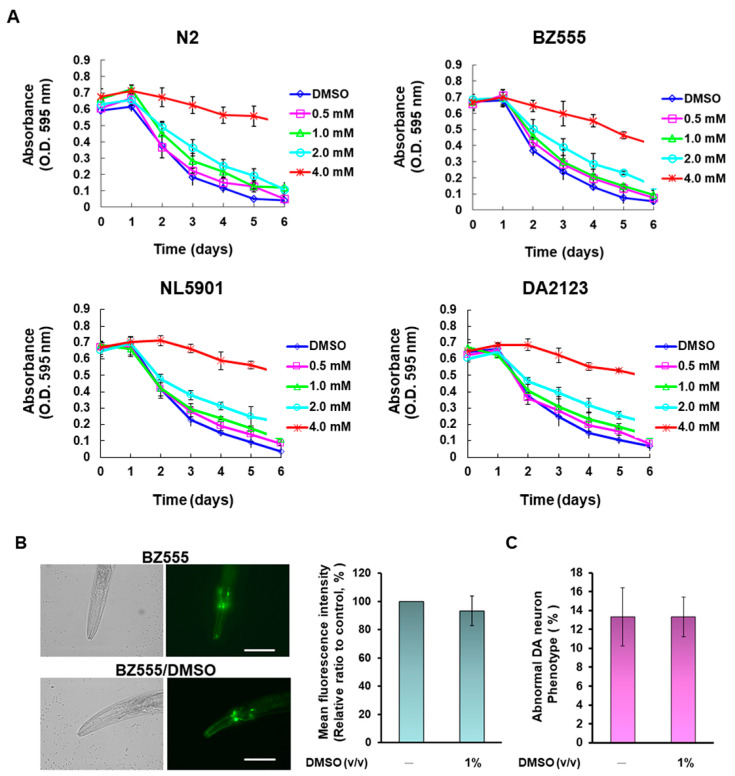

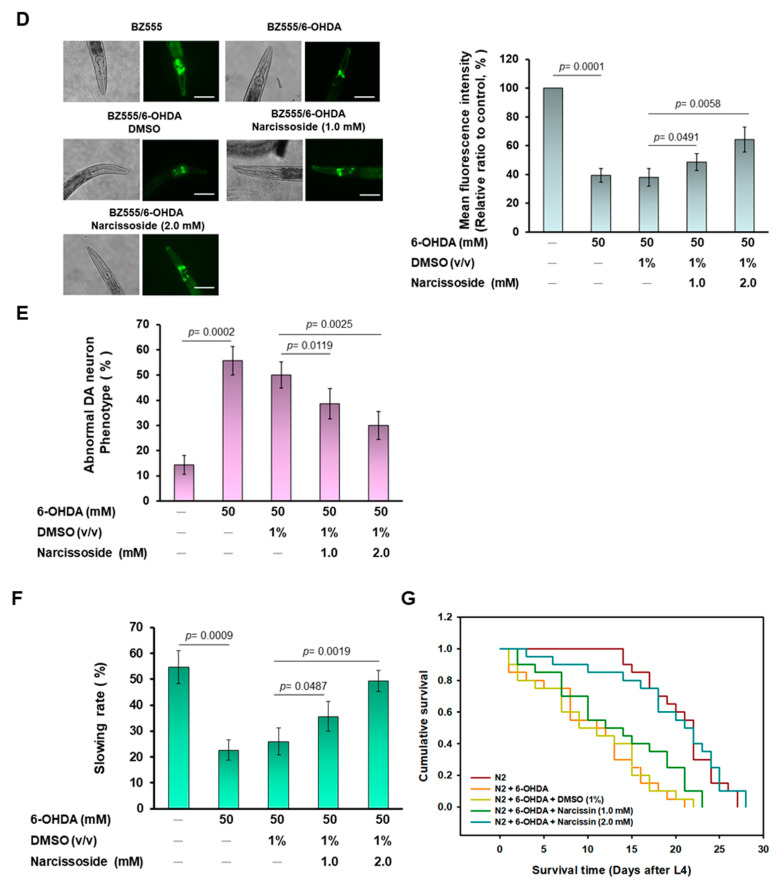
The 6-hydroxydopamine (6-OHDA)-caused dopaminergic (DA) neurons degeneration, food-sensitive behavioral deficits, and shortened lifespan in nematodes were ameliorated by narcissoside (NCS) pretreatment. (**A**) Appropriate concentrations of narcissoside (NCS) to treat nematodes were determined by food clearance assays. In 96-well plates, L1 stage nematodes of N2, BZ555, NL5901, and DA2123 were grown on S medium containing OP50 of *E. coli* (OD A_595_ = 0.6), and a series of dilution NCS concentrations. The plates were placed in an incubator at 20 °C for 6 days, and each well was recorded the OD value daily. (**B**,**C**) Transfer L1 stage nematodes of BZ555 to culture plates with or without DMSO (1%) and grow to L3 stage, following by exposure to 50 mM 6-OHDA for 1 h. Next, nematodes were transferred to 5-fluorodeoxyuridine (FUDR)-containing culture plates with or without DMSO (1%) and incubated for an additional 3 days for various analyses. (**B**) Representative GFP fluorescence images of DA neurons in the head of BZ555 nematodes in each group (left panel). Scale bar = 50 µm. The quantification of the fluorescence intensity of each group used ImageJ software (right panel). (**C**) BZ555 nematodes were scored for phenotypic deficits in DA neuron degeneration, with either disrupted axonal fluorescence signal or punctate as degeneration. Data are indicated as the percentage of nematodes with a DA neuron-deficient phenotype in each group. (**D**–**F**) Transfer L1 stage nematodes of BZ555 or N2 to culture plates with or without NCS and grow to L3 stage, following by exposure to 50 mM 6-OHDA for 1 h. Next, nematodes were transferred to 5-fluorodeoxyuridine (FUDR)-containing culture plates with or without NCS and incubated for an additional 3 days for various analyses. (**D**) Representative GFP fluorescence images of DA neurons in the head of BZ555 nematodes in each group (left panel). Scale bar = 50 µm. The quantification of the fluorescence intensity of each group used ImageJ software (right panel) (**E**) BZ555 nematodes were scored for phenotypic deficits in DA neuron degeneration, with either disrupted axonal fluorescence signal or punctate as degeneration. Data are indicated as the percentage of nematodes with a DA neuron-deficient phenotype in each group. (**F**) The ability of NCS to restore dopamine neuron function in 6-OHDA-exposed N2 nematodes was assessed using food-sensitive assay. The nematode was placed on a bacteria-free lawn and bacteria lawn to count separately the number of S-shaped movements within 20 s. Slowing rate (%) = (the number of S-shaped movements of the nematode on bacteria-free lawn—the number of S-shaped movements of nematode on bacteria lawn)/the number of S-shaped movements of nematode on bacteria-free lawn) ×100%. A total of 50 nematodes were counted in each group. (**G**) The lifespan of the NCS-pretreated 6-OHDA-exposed N2 nematodes was assessed by cumulative survival curves. The L3-stage nematodes were cultured in the plate with or without NCS, replaced with FUDR-containing fresh plates every three days. The number of surviving nematodes was counted daily until all nematodes died. The number of nematodes in each group was fifty.

To further evaluate the utility of NCS in animals, we used the widely used *C. elegans* model. First, we determined the optimal NCS treatment dose for this model using a food clearance assay. The results showed that the food clearance curves of the N2, BZ555, NL5901, and DA2123 strains were significantly slower when the medium was supplemented, with more than 4-mM NCS compared to the untreated group (Figure 9A). Additionally, nematode body size and number of offspring were significantly reduced under a dissecting microscope (data not shown). These data reflect that NCS above 4 mM is toxic to nematode feeding, growth, and reproduction. Since the food clearance curves of these four strains were not significantly inhibited below 2-mM NCS (Figure 9A), we treated the nematodes with up to 2-mM NCS in subsequent experiments.

Next, we assessed the protective capacity of NSCs on dopamine neurons damaged by 6-OHDA exposure in nematodes. The expression of green fluorescent protein (GFP) in DA neurons of BZ555 nematodes was analyzed by fluorescence microscopy, and the results showed that the fluorescence intensity and morphological defects ratio of three pairs of DA neurons in the head did not change after treatment of 1% DMSO (Figure 9B,C). However, fluorescence intensity had a significant drop due to 6-OHDA exposure, reflecting the damage and degeneration of DA neurons (Figure 9D, left panel). We quantified the fluorescence intensity using ImageJ software and revealed that the mean fluorescence intensity was diminished by 60.7% (*p* < 0.0001) in nematodes exposed to 6-OHDA compared with control nematodes (Figure 9D, right panel. However, NCS pretreatment dose-dependently restored GFP fluorescence intensity in 6-OHDA-exposed nematodes (Figure 9D, left panel). Pretreatment with 2 mM NCS elevated the fluorescence intensity 1.7-fold (*p* = 0.0058) compared to the NCS untreated 6-OHDA-exposed group (Figure 9D, right panel). Additionally, the ratio of abnormal phenotypes of DA neurons was significantly higher in 6-OHDA-exposed nematodes than in control nematodes by 3.9-folds (*p* = 0.0002) (Figure 9E). Pretreatment of 6-OHDA-exposed nematodes with 2 mM NCS significantly declined the phenotype of DA neuronal degeneration by 40.0% (*p* = 0.0025) compared with the unpretreated group (Figure 9E).

### 3.9. Narcissoside (NCS) Pretreatment Restores Deficits in Dopamine-Mediated Food-Sensitive Behavior in Nematodes Exposed to 6-OHDA

The function of DA neurons in nematodes is directly related to dopamine-mediated food-sensitive behavior [36]. When put in contact with food, the nematode reduces its speed of movement (the frequency with which the S-shaped body bends) to increase feeding efficiency. The results showed that wild-type N2 worms had a 54.7% slowing rate (quantitative representation of changes in bending frequency) after contact with bacterial lawns, compared to bacteria-free lawns (Figure 9F). Moreover, the slowing rate of N2 nematodes exposed to 6-OHDA was lessened by 58.5% (*p* = 0.0009) compared with the control group. However, this slowing rate was increased dose-dependently by NCS. Nematodes pretreated with 2 mM NCS experienced a 1.9-fold (*p* = 0.0019) increase in the slowing rate compared with the untreated group when exposed to 6-OHDA (Figure 9F). The above results confirm that NCS pretreatment can ease the functional deficits in DA neurons caused by 6-OHDA.

### 3.10. Narcissoside (NCS) Pretreatment Improves Shortened Lifespan of Nematodes Due to 6-OHDA Toxicity

Studies indicate that the average lifespan of patients with PD is shortened [37]. We therefore considered whether NCS could prolong the lifespan of nematodes affected by 6-OHDA toxicity. As revealed in Figure 9G, N2 nematodes exposed to 6-OHDA had shorter lifespans than control nematodes. Conversely, pretreatment with NCS dose-dependently prolongs lifespan in nematodes exposed to 6-OHDA (Figure 9G). A cumulative survival model estimated using the Kaplan–Meier method revealed that the mean survival time in the 6-OHDA-exposed group was 9.5 ± 1.4 days, which was significantly shorter compared to 19.9 ± 1.7 days in the control group (*p* < 0.0001). In contrast, the mean survival time of the 2-mM NCS-pretreated group after exposure to 6-OHDA was significantly longer than that of the unpretreated group (*p* < 0.0001), which was 18.5 ± 1.2 days (Figure 9G). Therefore, NCS pretreatment can ease the shortening of the lifespan caused by 6-OHDA.

### 3.11. Narcissoside (NCS) Pretreatment Declines the Level of Reactive Oxygen Species in 6-OHDA-Exposed Nematodes by Enhancing the GSH Production via Rising Expression of Skn-1, Gcs-1, and E01A2.1

**Figure 10:**
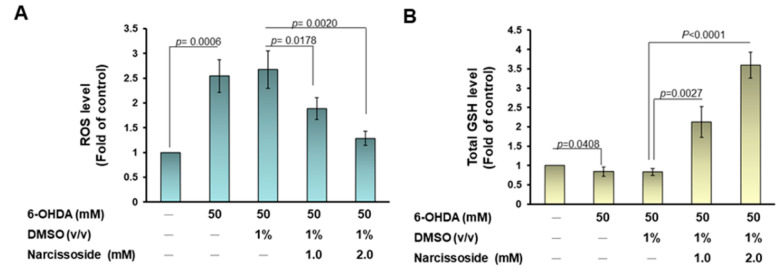

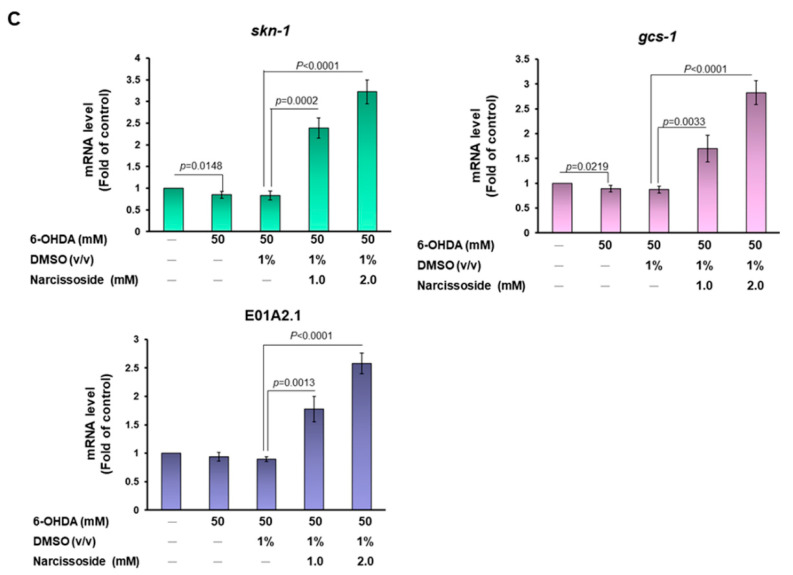
Pretreatment with narcissoside (NCS) significantly diminishes reactive oxygen species (ROS) in 6-OHDA-exposed nematodes by increasing the level of intracellular GSH by enhancing the mRNA expression of skin-1, gcs-1, and E01A2.1. L1 stage N2 nematodes were grown to L3 stage in medium containing NCS. It was then exposed to 6-OHDA for 1 h. Next, it was transferred to fresh plates containing NCS for an additional 3 days. (**A**) Thirty nematodes from each group were randomly selected to detect ROS levels in wells of a 96-well plate with the 2′,7′-dichlorodihydrofluorescein diacetate (H2DCFDA) probe. (**B**) GSH levels were measured in 100 randomly selected nematodes from each group using the GSH assay kit. (**C**) The mRNA expression of skin-1, gcs-1, and E01A2.1 in each group of N2 nematodes was quantified by qPCR.

Next, we wanted to assess whether NCS would improve ROS levels in nematodes exposed to 6-OHDA. Compared with controls, ROS levels in nematodes were significantly augmented by 2.5-fold (*p* = 0.0006) after exposure to 6-OHDA (Figure 10A). However, NCS dose-dependently reduced the level of ROS in nematodes exposed to 6-OHDA. 6-OHDA-induced ROS levels were lessened by 51.9% (*p* = 0.0020) after pretreatment with 2 mM NCS compared with the unpretreated group (Figure 10A). Taking further analysis, we found that NCS pretreatment dose-dependently increased the level of GSH in nematodes exposed to 6-OHDA. In the 2mM NCS pretreatment group, the level of GSH in 6-OHDA-exposed nematodes was augmented by 4.5-fold compared with the untreated group (*p* < 0.0001) (Figure 10B).

Next, we wanted to determine whether the neuroprotective effect of NCS in the nematode model is also enhanced by the activity of the Nrf2 transcription factor (The ortholog in *C. elegans* is skn-1), promoting the expression of GCLC (The ortholog in *C. elegans* is gcs-1) and GCLM (The ortholog in *C. elegans* is E01A2.1), thereby easing DA neuron apoptosis induced by 6-OHDA [38]. Analysis using real-time quantitative PCR (qPCR) indicated that NCS pretreatment dose-dependently raised the mRNA expression of skn-1, gcs-1, and E01A2.1 in 6-OHDA-exposed nematodes, respectively (Figure 10C). Under 6-OHDA exposure, the mRNA levels of skn-1, gcs-1, and E01A2.1 were elevated 4.0-fold (*p* < 0.0001), 3.1-fold (*p* < 0.0001) and 2.9-fold (*p* < 0.0001), respectively, in the group pretreated with 2 mM NCS compared with the unpretreated group (Figure 10C).

### 3.12. Narcissoside (NCS) Significantly Reduces α-Synuclein Accumulation by Promoting Autophagy of Nematodes

**Figure 11:**
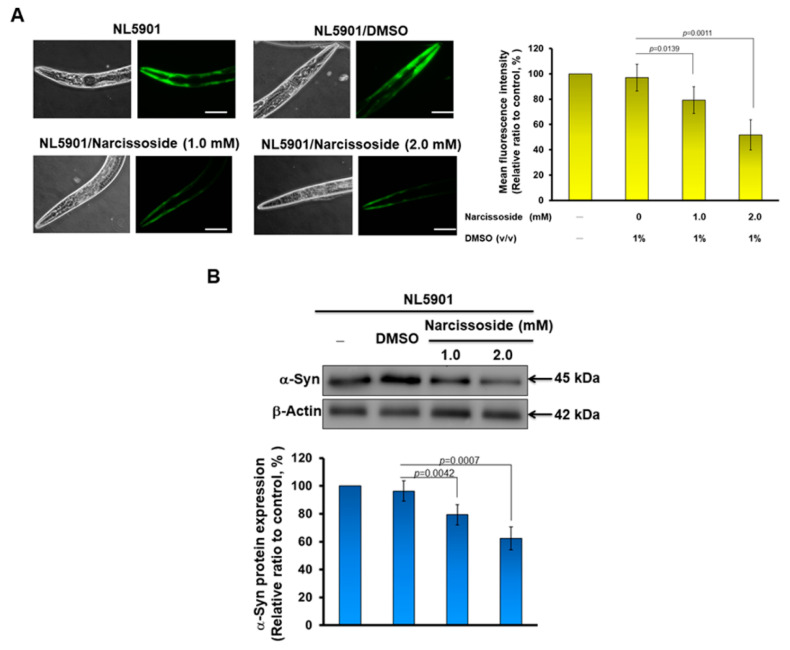

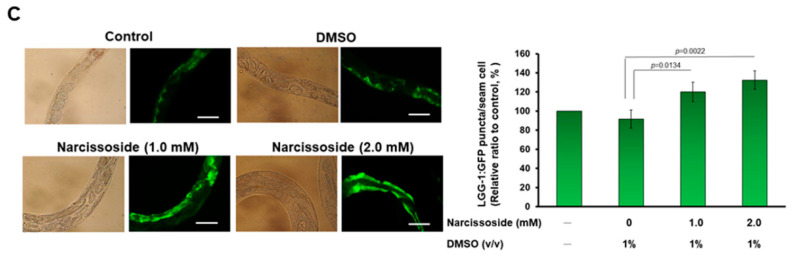
α-Synuclein accumulation in nematode muscle cells is diminished by narcissoside (NCS)-induced autophagy. (**A**) NL5901 nematodes at the L3 stage were treated with NCS for three days. The fluorescence intensity exhibited by accumulating α-synuclein in muscle cells was then analyzed using a fluorescence microscope (Leaf panel). The fluorescence intensity was quantified using ImageJ software (Right panel). (**B**) The expression of α-synuclein in each group of nematodes in (**A**) was analyzed by Western blotting. β-actin is an internal loading control (top panel). The fluorescence intensity was quantified using ImageJ software (bottom panel). (**C**) DA2123 nematodes at the L3 stage were treated with NCS for three days. The dots formed by the autophagy marker LGG-GFP in seam cells were observed (Leaf panel) and counted by a fluorescence microscope. The right panel is the average result of the fluorescent dot count analysis of all seam cells in each group of worms.

Abnormal accumulation of α-synuclein in dopamine neurons is a common hallmark of sporadic or hereditary PD. Therefore, we also wanted to investigate whether NCS treatment improved the ability for this abnormal accumulation. We used transgenic NL5901 nematodes, which have been widely used as an animal model of α-synuclein accumulation. Its muscle cells overexpress human α-synuclein protein, causing accumulation and developing PD-like movement disorders such as paralysis [39]. Our results of quantifying the fluorescence intensity of NL5901 worms in each group show that NCS treatment can dose-dependently reduce the accumulation of α-synuclein in muscle cells (Figure 11A left). In L3 stage NL5901 nematodes treated with 2 mM NCS for three days, we found a 46.7% reduction (*p* = 0.0011) in α-synuclein accumulation, compared with the untreated group (Figure 11A, right panel). We then used Western blotting to analyze the level of α-synuclein, which also showed a dose-dependent lessening in NCS. We found that after 2 mM of NCS treatment, the level of α-synuclein was reduced by 35.3% compared with the control group (Figure 11B).

Previous studies have confirmed that the activity of Nrf2 can positively regulate the function of autophagy [40]. Autophagy can be used to degrade abnormal protein accumulation in cells [41]. Therefore, we additionally used the transgenic worm strain DA2123 to evaluate the ability of NCS to induce autophagy [42]. The LGG-1 protein (orthology of human LC3) of its hypodermal seam cells was fused to GFP, and autophilic activity positively correlates with green fluorescent dots formed in seam cells. This is because autophagic activation recruits cytoplasmically dispersed LGG-1 binding and aggregates on autophagolysosomes, and thus serves as a tool for detecting autotrophic activity. The results show that NCS treatment can dose-dependently increase the activity of autophagy inworms (Figure 10C, left). Using 2 mM NCS to treat larvae at the L3 stage for three days, we found that the average number of green fluorescent dots per worm increased by 1.2 times compared with the control group (Figure 10C, right). The above results show that NCS treatment can increase the autophagy activity in worms.

## 4. Discussion

Many studies have confirmed that the process of neurodegenerative diseases such as PD is accompanied by oxidative stress and aging. Therefore, avoiding mitochondrial damage and ROS generation in DA neurons is a feasible direction for establishing the PD treatment strategy. In this study, we used 6-OHDA-exposed SH-SY5Y cells and *C. elegans* models to confirm that narcissoside (NCS) can lessen intracellular ROS and apoptosis, restore dopamine-mediated food-sensitivity behavior, and prolong lifespan in 6-OHDA-exposed nematodes. Moreover, NCS enhances autophagy activity and diminishes α–synuclein accumulation in nematodes. This is the first report of the neuroprotective capability of NCS.

Much evidence has shown that the antioxidant molecule glutathione (GSH) can prevent and treat DA neurodegeneration induced by various neurotoxins, such as 6-OHDA, MPTP, and rotenone [43]. Our further analysis found that the antioxidant property of NCS was also primarily achieved by raising the level of GSH in cells and nematodes. We used the inhibitor of γ-glutamylcysteine ligase (γ-GCL), buthionine sulphoximine, to block GSH synthesis in 6-OHDA-exposed SH-SY5Y cells, resulting in attenuated anti-oxidative and anti-apoptotic abilities of NCS. Conversely, SH-SY5Y cells treated with NAC, an enhancer of GSH production, eased ROS production and apoptosis induced by 6-OHDA exposure to a similar effect as NCS. These results indicate that NCS prevents ROS generation and apoptosis by regulating the GSH synthesis system. γ-GCL is the key enzyme in the step of GSH synthesis. Next, we found that the increase in the amount of intracellular GSH was due to the up-regulation of the GCL modifier subunit (GCLM, gcs-1is an ortholog in *C. elegans*) and GCL catalytic subunit (GCLC, *E01A2.1* is an ortholog in *C. elegans*).

The promoter regions of both GCLM and GCLC genes have antioxidant responsive element (ARE); therefore, we considered whether the activity of the ARE-specific transcription factor NF-E2-related factor 2 (Nrf2, *skn-1* is an ortholog in *C. elegans*) would be regulated by NCS [44]. Our study showed that 6-OHDA-exposed SH-SY5Y cells slightly increased the expression of Nrf2, which may be a phenomenon of the adaptive protective mechanism of the cells, itself triggered by the increase in ROS. Western blotting and ARE-associated electrophoretic mobility shift assay (EMSA) of nuclear extracts of SH-SY5Y showed that pretreatment with NCS augmented the amount of Nrf2 translocated into the nucleus. Furthermore, luciferase reporter assays also showed that NCS pretreatment enhanced the Nrf2/ARE-associated transcriptional activity. We subsequently used siRNA to downregulate the expression of Nrf2 in cells, and found that the inhibitory effect of NCS on 6-OHDA-induced ROS generation and apoptosis in SH-SY5Y cells were abolished. This result further confirms the critical role of Nrf2 in NCS-mediated neuroprotection.

Nrf2 is a basic leucine zipper (bZip) transcription factor, whose main function is to mediate the expression of genes related to cellular antioxidant and self-protection mechanisms [45]. Additionally, it is also involved in the regulation of mitochondrial metabolism, tissue repair, autophagy, ubiquitin-proteasome system (UPS), inflammation, and immune response, etc. [46]. Nrf2 possesses seven highly conserved Nrf2-ECH homology (Neh) domains. Among them, two Nehs are closely related to Nrf2 activity mediation. Neh2 is involved in the binding of Kelch like-ECH-associated protein 1 (Keap1, a to cytoplasmic inhibitor of Nrf2). The Neh6 domain contains a degron that regulates the rate of Nrf2 degradation, which prolongs the half-life of Nrf2 under stressful conditions. Nrf2 forms an inactive complex with Keap1 and cullin 3 in the cytoplasm under normal or unstressed conditions, and is rapidly degraded by proteasomes via ubiquitination [47]. Keap1 interacts with Nrf2 through a β-propeller structure formed by double glycine repeat (DGR) and C-terminal region (CTR) domains. Cysteine residues (Cys)151 in Bric-à-Brac/Tramtrack/Broad (BTB) domains and Cys273 and Cys288 in intervening region (IVR) domains assist Keap1 in sensing stress signals [48]. Cullin 3 is a core scaffold protein and combines with RING-box protein 1 (RBX1) and BTB protein to develop Cullin-RING E3 ubiquitin ligase complexes (CRLs). RBX1 recruits ubiquitin-conjugating enzyme (E2), and BTB is responsible for substrate recognition. CRLs are part of the UPS that add ubiquitin to lysine residues of targeted proteins, which are then carried to proteasomal degradation [49]. Cullin 3 ubiquitinates Nrf2 via the substrate adaptor protein Keap1. The ubiquitinated Nrf2 is then transported to the proteasome for degradation. Disruption of key cysteine residues in Keap1 by oxidative stress or electrophilic stress hinders the Keap1-Cul3 ubiquitination system, resulting in Nrf2 accumulation in the cytoplasm and translocation into the nucleus. Next, Nrf2 forms a heterodimer with Maf protein and binds to the ARE antioxidant response element in the promoter region of the target gene and recruits RNA polymerase for transcription.

In addition to genes of GCLC and GCLM, the downstream target genes of Nrf2 include NAD (P)H quinone oxidoreductase 1 (Nqo1), sulfiredoxin 1 (SRXN1), thioredoxin reductase 1 (TXNRD1), heme oxygenase-1 (HMOX1, HO-1) and glutathione S-transferase (GST), etc. [50]. Nqo1 has a detoxification function, which can reduce the two electrons of quinone to hydroquinone, indirectly preventing the single electron reduction in quinone to semiquinone free radical (ROS) [51]. SRXN1 is an endogenous antioxidant whose substrate is peroxiredoxin (Prxs) of S-hydroxy-S-oxocysteine and the product is Prxs- of S-hydroxycysteine. Prxs controls the levels and signaling of peroxides in cells. Therefore, SRXN1 can resist oxidative stress by restarting Prxs to maintain the balance between cellular oxidation and reduction [52]. TXNRD1 contains a selenocysteine (Sec) residue, reduces the oxidation state of substrates, such as thioredoxins in cells, and plays a role in preventing oxidative stress and maintaining selenium metabolism [53]. HO-1 catalyzes the degradation of heme to produce bilirubin; ferrous ions and carbon monoxide can resist endogenous oxidative stress, especially in the brain. HO-1 also participates in the signal transducer of carbon monoxide, with functions that include improving inflammation and hypoxic injury. Interestingly, although HO-1 promotes the release of ferrous ions from heme, Nrf2 upregulates ferritin gene transcription to increase ferrous storage and sequestration, thereby reducing labile iron to maintain iron homeostasis [54]. Some reports have shown that the disruption of iron homeostasis due to iron accumulation in dopamine neurons has been implicated in Parkinson’s disease [55]. GST catalyzes the modifying reaction of the reduced form of GSH to xenobiotic substrates in order to promote the solubility of xenobiotic substrates and prevent them from interacting with intracellular proteins and nucleic acids, to achieve the purpose of detoxification [56]. Therefore, the activation of Nrf2, in addition to increasing the amount of downstream GSH, should initiate multiple neuroprotective and antioxidative mechanisms, although these were not analyzed in this study.

6-OHDA-induced apoptosis is regulated by mitogen-activated protein kinase (MAPK) and phosphoinositide 3-kinases (PI3K)-Akt signaling pathways [57]. c-Jun NH2-terminal kinase (JNK) and p38 MAPK pathways have been revealed to be positive regulators of 6-OHDA-induced apoptosis [58,59]. In contrast, the extracellular signal-regulated kinase 1/2 (ERK) MAPK and PI3K/Akt pathways maintain cell survival and resist apoptosis [60]. This study found that NCS pretreatment reduced 6-OHDA-induced phosphorylation of JNK and p38, but promoted the activity of ERK and Akt signaling molecules. Here we find a question of whether NCS induces changes in the phosphorylation of these key signaling factors leading to Nrf2 activation, or if NCS promotes Nrf2 activation and induces changes in the activity of these molecules. Alternatively, NSC independently regulate the Nrf2 and MAPK/Akt pathways.

MAPK or Akt can phosphorylate Nrf2 at different sites to regulate Nrf2-mediated antioxidant responses [61]. Genetic defects and inhibition of p38 result in the upregulation of Nrf2-associated HO-1 gene expression [62]. MDA-7/IL-24 blocks Nrf2-mediated antioxidant response in order to induce apoptosis by activating the p38 pathway and inhibiting the ERK pathway [63]. Physalin A promotes Nrf2 phosphorylation and pathway activity in order to induce the expression of detoxification enzymes by upregulating ERK activity [64]. Agrimonolide and desmethylagrimonolide can significantly activate the ERK signaling pathway and attenuate p38 phosphorylation, activating Nrf2 by nuclear translocation [65]. However, studies have also shown that the genetic deletion of Nqo1 downstream of Nrf2 can activate JNK and p38 and enhance apoptosis [66]. The Nrf2-Srx-1 axis may protect cardiomyocytes by inhibiting mitochondria-dependent apoptosis by regulating the PI3K/AKT pathway [67]. Phenylallylidenecyclohexenone analogs inhibit Nrf2-associated TXNRD1 activity, then significantly activate p38 signaling and inhibit Akt/mTOR signaling [68]. To confirm the causal relationship between Nrf2 activation and MAPK/Akt phosphorylation under NCS treatment, we performed RNAi of Nrf2 and MAPK inhibitor treatment experiments, respectively. The results showed that the downregulation of Nrf2 expression reversed the ability of NCS to reduce JNK and P38 activity in 6-OHDA-exposed SH-SY5Y cells. Similarly, the ability of NCS to increase ERK and Akt phosphorylation was also abolished. However, treatment with inhibitors of JNK (SP600125) or P38 (SB 202190) did not abrogate the upregulation of Nrf2 activity under NCS treatment (data not shown). It is obvious that NCS mainly activate Nrf2 activity first to regulate the phosphorylation of MAPK and Akt. The bridge of this connection may be caused by Nrf2-mediated transcription of genes related to antioxidants and neuroprotection to reduce 6-OHDA-induced oxidative stress.

Furthermore, we explored the detailed mechanism by which NCS activates Nrf2. Since Nrf2 is tightly bound and induced to degradation by keap1/cullin 3 in the cytoplasm, we wanted to see whether NSC treatment disrupted their interactions. However, a yeast two-hybrid-based growth assay [35] showed that NSC did not disrupt their interaction (data not shown). However, using Western blotting, we found that Keap1 protein expression was significantly reduced by NSC treatment. Previous studies have shown that Keap1 expression is inhibited by multiple miRs, including miR-200a [69,70,71,72,73,74,75], miR-7a [76], miR-24-3p [77], miR-26b [78], miR-29 [79], miR-30a-3p [80], miR-34b [81], miR-125b [82,83], miR-141 [84,85], miR-223 [86], miR-328-3p [87], miR-421 [88], miR-432 [89], miR-512-3p [90], miR-626 [91], miR-941 [92], and miR-1225 [93]. They were all further analyzed for quantitative changes under NCS treatment. The results indicated that only miRNA-200a was significantly increased by NCS treatment. Moreover, Nrf2-mediated transcriptional activation induced by NSCs was significantly reversed by anti-miR-200a (miR-200a inhibitor) treatment. Therefore, our results confirmed that NCS can improve 6-OHDA-induced ROS stress of SH-SY5Y cells via rising miRNA-200a/Nrf2 axis and thereby inhibits the apoptosis-related JNK/P38 pathway and activates cell survival-associated phosphorylation of ERK and Akt. Incidentally, p62/SQSTM1 has also been shown to act as a competitive inhibitor of Keap1 binding to Nrf2 [94]. However, we did not find an increase in p62 expression and phosphorylation under NCS treatment (data not shown).

Some compounds increase cellular Nrf2 activity or increase GSH levels to achieve anti-oxidative stress and neuroprotective effects. Resveratrol pretreatment-induced intracellular Nrf2 phosphorylation, GSH and phase 2 enzymes in a 6-OHDA-injected PD rat model, thereby reducing ROS-induced DA neuronal apoptosis; promoting mitochondrial activity; restoring proteasome system function; and reducing inflammation, ER stress and behavioral/cognitive impairments [95]. Disubstituted dithiolethione ACDT [96], chrysin [97], 7,8,4′-trihydroxyisoflavone [17], etc., also have this property. Additionally, several phytocompounds have anti-apoptotic potential by modulating MAPK and Akt activities. γ-Mangostin inhibits p38 phosphorylation, and reduces intracellular ROS production and the caspase 3 activity, thereby restoring neuronal activity that has been damaged by 6-OHDA [98]. Compounds that form Dihuang Granule can reduce nigrostriatal pathway apoptosis and increase TH neuron number in 6-OHDA-induced PD rat models by inhibiting JNK/AP-1 pathway [99]. In this study, we found that NCS has similar antioxidant and anti-apoptotic mechanisms of action to these compounds. Moreover, we indicated that NCS improves the accumulation of α-synuclein in nematode muscle cells. The Nrf2 pathway drives oxidative stress-induced autophagy to protect cells from damage [100]. Our study also found that NCS enhanced autophagic activity in nematodes, possibly resulting in clearance of α-synuclein accumulation.

Diabetes and inflammation are known risk factors for inducing PD, and the NSC has the potency to ease these factors [101]. It is worth mentioning that recent studies have shown that NSCs can fight against COVID-19 [26]. The susceptibility of the elderly to COVID-19 infection, and the high mortality rate, are strongly associated with indirect clinical outcomes in PD patients [102]. Therefore, compared with the other above-mentioned compounds, NCS has more advantages in the protective potential of anti-PD.

## 5. Conclusions

According to our experimental results, phytochemical NCS can prevent ROS-induced oxidative stress and apoptosis in the SH-SH5Y cell and nematode models by inducing the MiR-200a/Nrf2/GSH axis, and inhibit the JNK and p38 MAPK pathways, while enhancing the phosphorylation of ERK and Akt molecules. Additionally, NCS also improved nematode α-synuclein accumulation by activating autophagy. Therefore, NCS can be developed as an anti-PD, or other neurodegenerative therapeutic agents, which deserves further evaluation in mammalian models and neurons derived from patient-induced pluripotent stem cells.

## Figures and Tables

**Table 1:** Primers for qPCR.

Genes of *C. elegans* (Human)	Primer Sequences (5′-3′)	(Start→End) Size (bp)
Skn-1 (Nrf-2)	Forward: 5′-TCCACCAGCATCTCCATTC-3′ Reverse: 5′-ACTTCTCCATAGCACATCAATC-3′	(480–600) 121
gcs-1 (Gclc)	Forward: 5′-GTTACAAGCCGAAGAGCAG-3′ Reverse: 5′-TGAAGCAGCGATGGACC-3′	(231–361) 131
E01A2.1 (Gclm)	Forward: 5′-CACCAATCCAAACCTCTACTC-3′ Reverse: 5′-TCAAAAGTGGCAGCAATAGC-3′	(882–1019) 138

## Data Availability

The data are contained within the article.
